# Respirable stone particles differ in their ability to induce cytotoxicity and pro-inflammatory responses in cell models of the human airways

**DOI:** 10.1186/s12989-021-00409-y

**Published:** 2021-05-06

**Authors:** Vegard Sæter Grytting, Magne Refsnes, Johan Øvrevik, Marit Sigrid Halle, Jasmin Schönenberger, Roelant van der Lelij, Brynhild Snilsberg, Tonje Skuland, Richard Blom, Marit Låg

**Affiliations:** 1 Section of Air Pollution and Noise, Department of Environmental Health, Domain of Infectious Disease Control and Environmental Health, Norwegian Institute of Public Health, PO Box 4404, Nydalen, N-0403 Oslo, Norway; 2 Geological Survey of Norway, Trondheim, Norway; 3 Norwegian Public Roads Administration, Trondheim, Norway; 4 SINTEF Industry, Oslo, Norway

**Keywords:** Particulate matter, Mineral particles, Stone particles, Quartz, Silica, Macrophages, Epithelial cells, Inflammation, NLRP3 inflammasome

## Abstract

**Background:**

Respirable stone- and mineral particles may be a major constituent in occupational and ambient air pollution and represent a possible health hazard. However, with exception of quartz and asbestos, little is known about the toxic properties of mineral particles. In the present study, the pro-inflammatory and cytotoxic responses to six stone particle samples of different composition and with diameter below 10 μm were assessed in human bronchial epithelial cells (HBEC3-KT), THP-1 macrophages and a HBEC3-KT/THP-1 co-culture. Moreover, particle-induced lysis of human erythrocytes was assessed to determine the ability of the particles to lyse biological membranes. Finally, the role of the NLRP3 inflammasome was assessed using a NLRP3-specific inhibitor and detection of ASC oligomers and cleaved caspase-1 and IL-1β. A reference sample of pure α-quartz was included for comparison.

**Results:**

Several stone particle samples induced a concentration-dependent increase in cytotoxicity and secretion of the pro-inflammatory cytokines CXCL8, IL-1α, IL-1β and TNFα. In HBEC3-KT, quartzite and anorthosite were the most cytotoxic stone particle samples and induced the highest levels of cytokines. Quartzite and anorthosite were also the most cytotoxic samples in THP-1 macrophages, while anorthosite and hornfels induced the highest cytokine responses. In comparison, few significant differences between particle samples were detected in the co-culture. Adjusting responses for differences in surface area concentrations did not fully account for the differences between particle samples. Moreover, the stone particles had low hemolytic potential, indicating that the effects were not driven by membrane lysis. Pre-incubation with a NLRP3-specific inhibitor reduced stone particle-induced cytokine responses in THP-1 macrophages, but not in HBEC3-KT cells, suggesting that the effects are mediated through different mechanisms in epithelial cells and macrophages. Particle exposure also induced an increase in ASC oligomers and cleaved caspase-1 and IL-1β in THP-1 macrophages, confirming the involvement of the NLRP3 inflammasome.

**Conclusions:**

The present study indicates that stone particles induce cytotoxicity and pro-inflammatory responses in human bronchial epithelial cells and macrophages, acting through NLRP3-independent and -dependent mechanisms, respectively. Moreover, some particle samples induced cytotoxicity and cytokine release to a similar or greater extent than α-quartz. Thus, these minerals warrant further attention in future research.

**Supplementary Information:**

The online version contains supplementary material available at 10.1186/s12989-021-00409-y.

## Background

The earth’s crust is composed of minerals, primarily in the form of silicates such as feldspars, quartz, pyroxenes, amphiboles, micas and clay minerals [1]. Several rocks and minerals are excavated for use in a range of applications, including construction, ceramics, paints, fillers, abrasives, plastics and electronics [2]. Thus, inhalation of respirable mineral particles is a potential health hazard in industries and occupations where rocks and minerals are mined, processed and handled [3–6]. In addition, mineral particles may be a major constituent in ambient particulate matter (PM), stemming from both anthropogenic activities and from natural sources [7–9]. Mineral-rich particles generated by road wear are especially important in northern countries due to the prevalent use of studded tyres during the winter and spring seasons, which increases the contribution from road surface abrasion [10–12].

Mineral particles are more prevalent in the coarse fraction of ambient PM with an aerodynamic diameter between 10 μm and 2.5 μm (PM_10–2.5_) [13, 14]. Although health effects studies have predominately focused on particles with an aerodynamic diameter less than 2.5 μm or 10 μm (PM_2.5_ and PM_10_), the available epidemiological literature also suggests an association between ambient coarse particles and mortality and hospitalisations due to respiratory and cardiovascular diseases [15–21]. However, to what extent the mineral fraction contributes to the health effects of coarse PM is currently unknown. On the other hand, the impact of mineral particles on the development of respiratory disease is well known from occupational settings. Long-term occupational exposure to crystalline silica or asbestos particles is linked to silicosis and asbestosis, conditions characterized by persistent inflammation and pulmonary fibrosis, and may lead to development of lung cancer [22–24].

Experimental studies in vivo suggest that the inflammatory and fibrotic effects of crystalline silica are driven by the reactive surface of the particles and are mediated by activation of the nucleotide-binding oligomerization domain (NOD)-like receptor containing pyrin domain 3 (NLRP3) inflammasome [25–27]. Activation of NLRP3 has also been reported for several other crystalline and non-crystalline particulate compounds, such as asbestos fibres, gout-associated monosodium urate (MSU) and calcium pyrophosphate dihydrate crystals (CPPD), cholesterol crystals and nanoparticles [28–33]. After particle phagocytosis, the reactive surface of crystalline silica is hypothesized to destabilize the phagolysosomal membrane, causing leakage of lysosomal content and the subsequent activation of NLRP3 [30, 34–37]. In line with the central role of lysosomal destabilization, particle-induced hemolysis has been used as an in vitro measure of membranolytic properties and has been shown to predict the toxicity of silica particles [27, 35]. Upon activation, NLRP3 oligomerises and recruits apoptosis-associated speck-like protein containing a CARD (ASC) and pro-caspase-1, forming a large multi-protein complex. This leads to auto-activation of caspase-1, which subsequently generates the active form of the pro-inflammatory cytokines IL-1β and IL-18 through proteolytic cleavage [38]. In addition to IL-1β, IL-1α has been reported to be a central cytokine in the response to inhaled quartz particles and may promote the expression of IL-1β [39]. NLRP3 and caspase-1 have also been implicated in IL-1α processing and secretion, although their roles are not fully clarified [40–42]. IL-1α and IL-1β are potent pro-inflammatory cytokines that bind to interleukin-1 receptor 1 (IL-1R1) and activate the inflammatory response in the recipient cell through transcription factors such as activator protein (AP)-1 and nuclear factor kappa B (NFκB) [43]. Induction of pulmonary inflammatory responses is considered a key event in PM-induced diseases, with pulmonary epithelial cells and macrophages being among the primary targets [44].

While many studies have assessed the effects of silica and asbestos particles, less is known regarding the toxicity of other stone- and mineral particles. Previous studies from our group have shown that stone particles of different mineral and elemental composition differ in their ability to induce inflammatory cytokines both in vitro and in vivo [45–51]. Although the mechanisms were not fully identified, differences between particle samples could not be attributed to particle-induced reactive oxygen species (ROS) or soluble metal constituents, suggesting that characteristics of the insoluble fraction were responsible [45, 49]. The present study expands this work and compares six different stone particle samples of different mineral composition from Norwegian stone quarries. To assess the potential toxicity of the stone particles in the human airways, the ability to induce cytotoxicity and secretion of pro-inflammatory cytokines was explored in human bronchial epithelial cells and THP-1-derived macrophages, and a co-culture of epithelial cells and THP-1 macrophages. Furthermore, the ability of the particles to induce lysis of human red blood cells was assessed to explore membranolytic properties. Finally, the role of the NLRP3 inflammasome in the observed effects was investigated using a small molecule inhibitor against NLRP3 and western blot analysis of key events in NLRP3-mediated inflammation.

## Results

### Particle characteristics

#### Particle sample composition

**Fig. 1:**
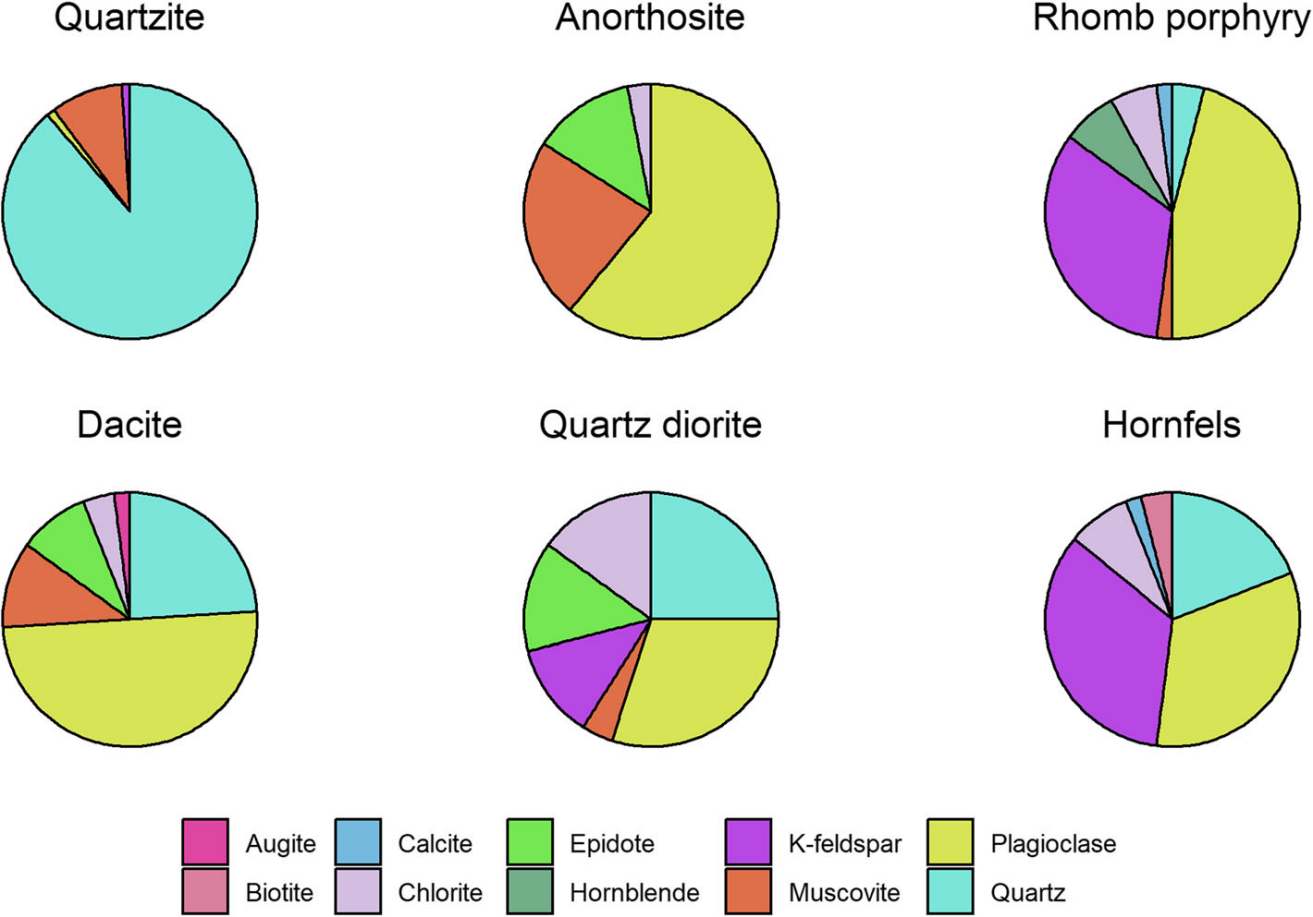
Stone particle mineral composition. The mineral composition of the quartzite, anorthosite, rhomb porphyry, dacite, quartz diorite and hornfels samples was determined by XRD analysis and is presented as percentages

The mineralogical composition of the stone particle samples, as determined by X-ray diffraction (XRD) analysis, is presented in Fig. 1. The different samples varied considerably in their mineral composition. Quartz and feldspar minerals, in the form of plagioclase or K-feldspar, were the most common mineral constituents, while muscovite, epidote, biotite, hornblende, calcite and chlorite were present in smaller amounts in some of the samples. The quartzite sample consisted primarily of quartz in addition to small amounts of feldspar and muscovite. The rhomb porphyry and anorthosite samples, on the other hand, consisted mainly of feldspar minerals, as well as other minerals such as epidote, chlorite, muscovite, calcite, quartz, hornblende and calcite. Notably, anorthosite contained over 20% muscovite, the largest amounts of all the samples. The dacite, quartz diorite and hornfels samples contained both quartz and feldspar minerals to a varying degree, in addition to smaller amounts of other minerals. The elemental composition of the particles was determined using X-ray fluorescence (XRF) analysis and is presented in Table S1 (online supplementary materials). SiO_2_ and Al_2_O_3_ were the major components in all particle samples. In addition, all samples contained varying amounts of Fe_2_O_3_, CaO, K_2_O, MgO, Na_2_O, SrO, P_2_O_5,_ TiO_2_ and BaO. Cr_2_O_3_, CuO, NiO, V_2_O_3_, ZnO, ZrO_2_ were also present in one or more of the samples.

#### Particle size and surface area

The particle size distributions for the stone particle samples are presented in Table 1. The samples consisted of a mixture of differently sized particles with substantial amounts of particles ranging from 10 to 1 μm. For all samples, over 90% of the particles were below 10 μm in diameter. The quartzite and anorthosite samples contained the highest amounts of large particles, with 7.6 and 4.6% of the samples being over 10 μm, respectively. In comparison, the hornfels sample and the α-quartz reference sample contained the smallest particles with approximately 50% being below 2.5 μm. The dacite, rhomb porphyry and quartz diorite samples displayed similar size distributions.

**Table 1:** Particle size distribution. The size distribution of the particles was determined by coulter counter analysis and is presented as percent particles in the size ranges < 1, 1–2.5, 2.5–5, 5–10 and > 10 μm

Diameter (μm)	Quartzite	Anorthosite	Rhomb porphyry	Dacite	Quartz diorite	Hornfels	α-quartz
< 1	0.5	4.1	4.7	2.7	1.4	7.1	4.5
1–2.5	24.2	26.0	36.0	32.1	30.5	43.4	43.6
2.5–5	29.7	30.7	33.6	32.7	33.2	32.8	40.0
5–10	37.9	34.6	25.0	30.6	32.8	16.6	11.9
> 10	7.6	4.7	0.8	2.1	2.2	0.0	0.0

The specific surface areas of the particle samples were determined by Brunauer-Emmett-Teller (BET) analysis (Table 2). The quartzite sample had the smallest surface area of 3.8 m^2^/g, followed by quartz diorite and dacite with surface areas of 5.1 and 5.2 m^2^/g. Hornfels had a surface area of 6.0 m^2^/g, while the α-quartz, anorthosite and rhomb porphyry samples had the largest surface areas of 7.0, 7.2 and 7.2 m^2^/g, respectively.

**Table 2:** Particle surface area

Particle sample	Particle surface area (m^2^/g)
Quartzite	3.8
Anorthosite	7.2
Rhomb porphyry	7.2
Dacite	5.2
Quartz diorite	5.1
Hornfels	6.0
α-quartz	7.0

#### Endotoxin contamination

Studies suggest that bacterial endotoxin can contribute to particle-induced inflammation [52]. Thus, the levels of endotoxin in the particle samples were determined based on the amebocyte lysate method. In general, low levels of endotoxin below 0.15 EU/mg were detected (Table 3). The quartzite and hornfels samples had the highest levels, followed by rhomb porphyry, dacite and quartz diorite. The content of endotoxin in the anorthosite sample and the α-quartz reference sample was below the limit of detection for the assay. Importantly, no significant positive correlations were detected between endotoxin content and the biological endpoints described in the subsequent sections.

**Table 3:** Endotoxin content

Particle sample	Endotoxin content (EU/mg)
Quartzite	0.127
Anorthosite	–
Rhomb porphyry	0.101
Dacite	0.097
Quartz diorite	0.076
Hornfels	0.132
α-quartz	–

#### Binding of cytokines to particles

Studies report that particles may bind secreted cytokines non-specifically, possibly confounding the results of bioassays such as enzyme-linked immunosorbent assay (ELISA) [53]. To determine if this was the case for the stone particle samples used in the present study, cell-free solutions of CXCL8, IL-1α, IL-1β and TNFα were incubated with 400 μg/mL stone particles for 24 h, after which the cytokines remaining in the medium was determined by ELISA. The results show that while all particle samples bound relatively low levels of cytokines in the RPMI medium used in experiments with the THP-1 macrophages, the stone particles bound relatively large amounts of CXCL8 and IL-1β in the DMEM medium used with the HBEC3-KT cells (Figure S1). While the particle samples had approximately the same ability to bind IL-1β, the anorthosite particles bound larger amounts of CXCL8 compared with the rest of the stone particle samples (Figure S1). Conversely, the α-quartz reference sample only bound low levels of both cytokines (Figure S1).

### Cell viability

**Fig. 2:**
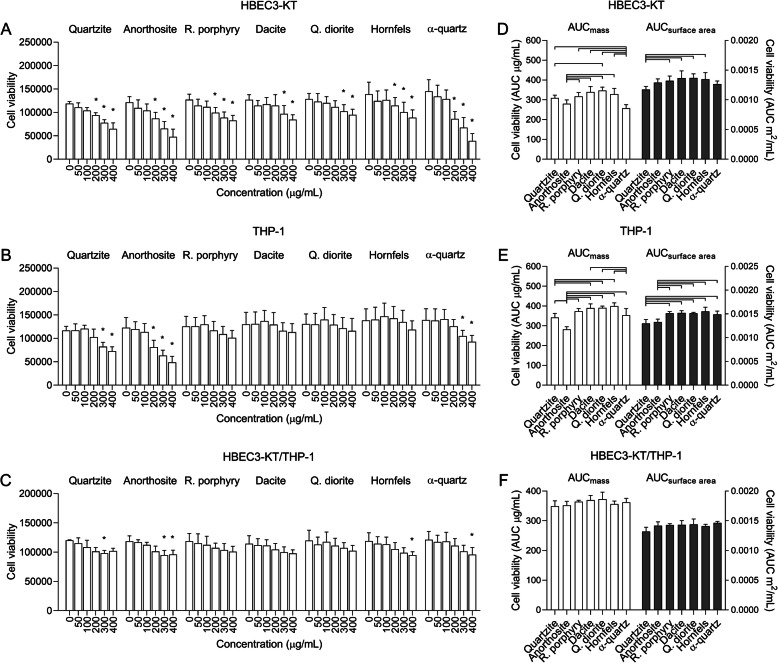
Exposure to stone particles causes sample- and concentration-dependent decreases in cell viability. HBEC3-KT cells (**a** and **d**), THP-1 macrophages (**b** and **e**), and a co-culture HBEC3-KT cells and THP-1 macrophages (**c** and **f**) were exposed to 0–400 μg/mL quartzite, anorthosite, rhomb porphyry, dacite, quartz diorite, hornfels and α-quartz for 24 h. Cell viability was determined by alamarBlue© assay. Results are presented as mean ± SD (*n* = 4–8). Area under the curve (AUC) values in (**d**-**f**) were calculated from the values in (**a**-**c**) for the concentration-range of 0–400 μg/mL and are presented related to equal mass (white bars) and surface area (grey bars). All values were normalized to account for differences in baseline prior to calculating AUC values by dividing each value by its respective control. Statistical significance is based on a two-way ANOVA followed by Dunnet’s post-test (**a**-**c**) or a one-way ANOVA followed by Tukey post-test (**d**-**f**). Based on deviations from normality and homoscedasticity, the data in (**b** and **c**) were log-transformed prior to statistical analysis. Asterisks (*) indicate statistically significant difference from the respective control (**a**-**c**), while a capped line indicates statistically significant difference between samples (**d**-**f**)

The effects of particle exposure on cell viability, as measured by alamarBlue™ assay, differed between particle samples and cell models (Fig. 2). In HBEC3-KT cells, all samples induced a concentration-dependent decrease in cell viability that was significantly different from control at 200–300 μg/mL (Fig. 2a). Area under the curve (AUC) values were calculated to compare the responses between the different particle samples. When the AUC values were calculated based on exposure on an equal mass basis, α-quartz was significantly more cytotoxic than all particle samples except anorthosite (Fig. 2d). The anorthosite sample was the second most cytotoxic, causing significantly higher reductions in cell viability than rhomb porphyry, dacite, quartz diorite and hornfels (Fig. 2d). In addition, the quartzite sample was significantly more cytotoxic than quartz diorite (Fig. 2d). Based on the results of the BET analysis, AUC values were then adjusted for differences in particle surface area by changing the concentration-metric for each particle sample to the equivalent concentration in m^2^/mL before fitting a curve to the values and calculating new AUC values from the portion of the curve that was common to all the particles. As the samples exhibited different specific surface areas the concentration-range for each particle sample was truncated to a different degree. The adjusted AUC values corresponded to mass-based concentration ranges of 0–400 μg/mL for quartzite, 0–211 μg/mL for anorthosite and rhomb porphyry, 0–292 μg/mL for dacite, 0–298 μg/mL for quartz diorite, 0–253 μg/mL for hornfels and 0–217 μg/mL for α-quartz. After adjusting for differences in surface area, quartzite was the most cytotoxic particle sample and caused a significantly larger reduction in cell viability compared to rhomb porphyry, dacite, quartz diorite and hornfels, while no significant difference could be detected between the remaining samples (Fig. 2d).

Compared with HBEC3-KT cells, most particle samples were less cytotoxic in THP-1 macrophages (Fig. 2b). Anorthosite, quartzite and α-quartz caused significant reductions in cell viability at 200–300 μg/mL compared to their respective control, while no significant reductions were induced by the remaining particle samples. When comparing AUC values at an equal mass basis, anorthosite caused significantly greater reductions in cell viability compared to all other samples, while quartzite and α-quartz were more cytotoxic than dacite, quartz diorite and hornfels (Fig. 2e). However, when comparing responses at equal surface area concentrations, quartzite and anorthosite were the most cytotoxic, causing similar reductions in cell viability that were significantly greater than all other particle samples (Fig. 2e).

Growing HBEC3-KT cells and THP-1 macrophages together in a co-culture seemed to protect against some of the cytotoxic effects of the particles (Fig. 2c). The quartzite, anorthosite, hornfels and α-quartz samples caused similar reductions in cell viability reaching statistical significance at 300, 300, 400 and 400 μg/mL, respectively (Fig. 2c). However, no significant differences were detected between the particle samples when comparing AUC values, either at equal mass or surface area (Fig. 2f).

### Release of pro-inflammatory cytokines

**Fig. 3:**
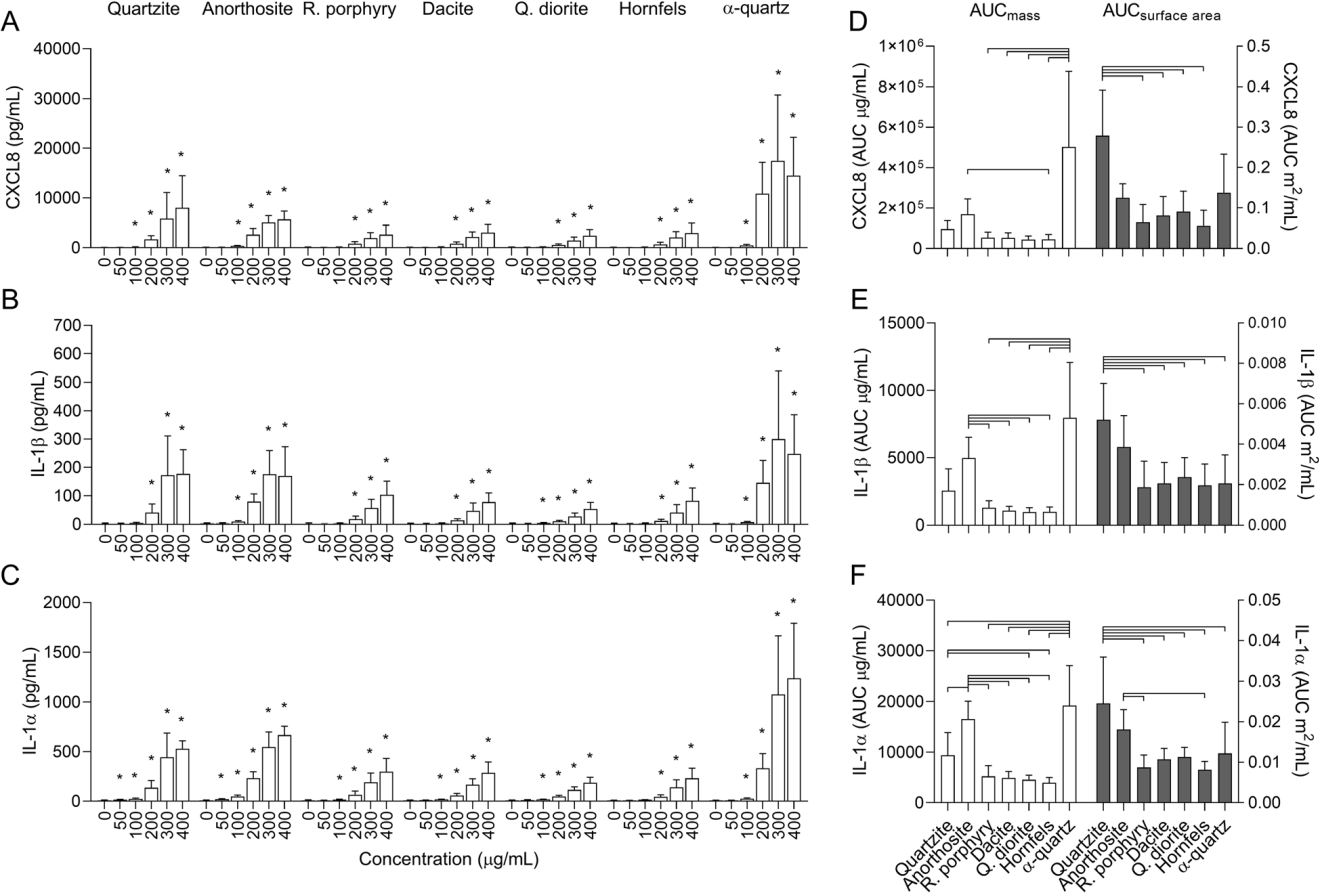
Stone particles induce sample- and concentration-dependent increases of pro-inflammatory cytokines in HBEC3-KT cells. HBEC3-KT cells were exposed to 0–400 μg/mL quartzite, anorthosite, rhomb porphyry, dacite, quartz diorite, hornfels and α-quartz for 24 h. The release of CXCL8 (**a** and **d**), IL-1β (**b** and **e**) and IL-1α (**c** and **f**) in the cell culture supernatant was measured by ELISA. Results are presented as mean ± SD (*n* = 5–7). Area under the curve (AUC) values in (**d**-**f**) were calculated from the values in (**a**-**c**) for the concentration-range of 0–200 μg/mL, and are presented related to equal mass (white bars) and surface area (grey bars). Statistical significance is based on a two-way ANOVA followed by Dunnet’s post-test (**a**-**c**) or a one-way ANOVA followed by Tukey post-test (**d**-**f**). Except for adjusted values in (**e**), the data in (**a**-**f**) were log-transformed prior to statistical analysis based on deviations from normality and homoscedasticity. Asterisks (*) indicate statistically significant difference from the respective control (**a**-**c**), while a capped line indicates statistically significant difference between samples (**d**-**f**)

Inflammatory reactions are considered central to the adverse health effects from mineral particle exposure. Thus, the particle-induced secretion of pro-inflammatory cytokines was measured in the cell culture supernatants by ELISA. All stone particle samples induced concentration-dependent increases in CXCL8, IL-1β and IL-1α secretion in HBEC3-KT cells, and a similar order of potency between samples was detected for all cytokines (Fig. 3). The α-quartz reference sample induced the highest amounts, followed by quartzite and anorthosite, while rhomb porphyry, dacite, quartz diorite and hornfels induced similarly low levels. As described, several of the particle samples induced considerable reductions in cell viability (Fig. 2a and d), which likely affected the cytokine responses at the highest concentrations. To avoid underestimation of the pro-inflammatory potential of the most cytotoxic samples, AUC values based on the concentrations of 0–200 μg/mL were used for the between-sample comparisons (Fig. 3d-f). As described above, AUC values reflecting exposure at equal surface area were estimated by transforming the concentrations to m^2^/mL, before fitting a curve to the values and estimating new values for the concentration-range that was common to all the particle samples. The surface area-adjusted AUC values corresponded to mass-based concentrations of 0–200 μg/mL for quartzite, 0–106 μg/mL for anorthosite and rhomb porphyry, 0–146 μg/mL for dacite, 0–149 μg/mL for quartz diorite, 0–127 μg/mL for hornfels and 0–109 μg/mL for α-quartz. At an equal mass basis, anorthosite and α-quartz were the most potent and induced significantly higher levels of IL-1α than all the other particle samples and higher IL-1β responses than all samples except quartzite (Fig. 3e and f). The α-quartz sample also induced a significantly higher CXCL8 response than rhomb porphyry, dacite, quartz diorite and hornfels, while the response induced by the anorthosite sample was only greater than hornfels (Fig. 3d). Quartzite tended to induce intermediate levels of all cytokines, but the effects were mostly non-significant when compared to the other particle samples. Adjusting AUC values for differences in surface area increased the effect of quartzite relative to the other samples for all cytokines, while the effects of anorthosite and α-quartz were diminished. At equal surface area, quartzite was the most potent particle sample and induced higher levels of CXCL8 than rhomb porphyry, dacite, quartz diorite and hornfels, and higher levels of IL-1β and IL-1α than all samples except anorthosite (Fig. 3d-f). Although the anorthosite sample induced intermediate levels of IL-1β and IL-1α, the differences were mostly non-significant (Fig. 3e and f). No significant differences were detected between the remaining particle samples for any cytokine (Fig. 3d-f).

**Fig. 4:**
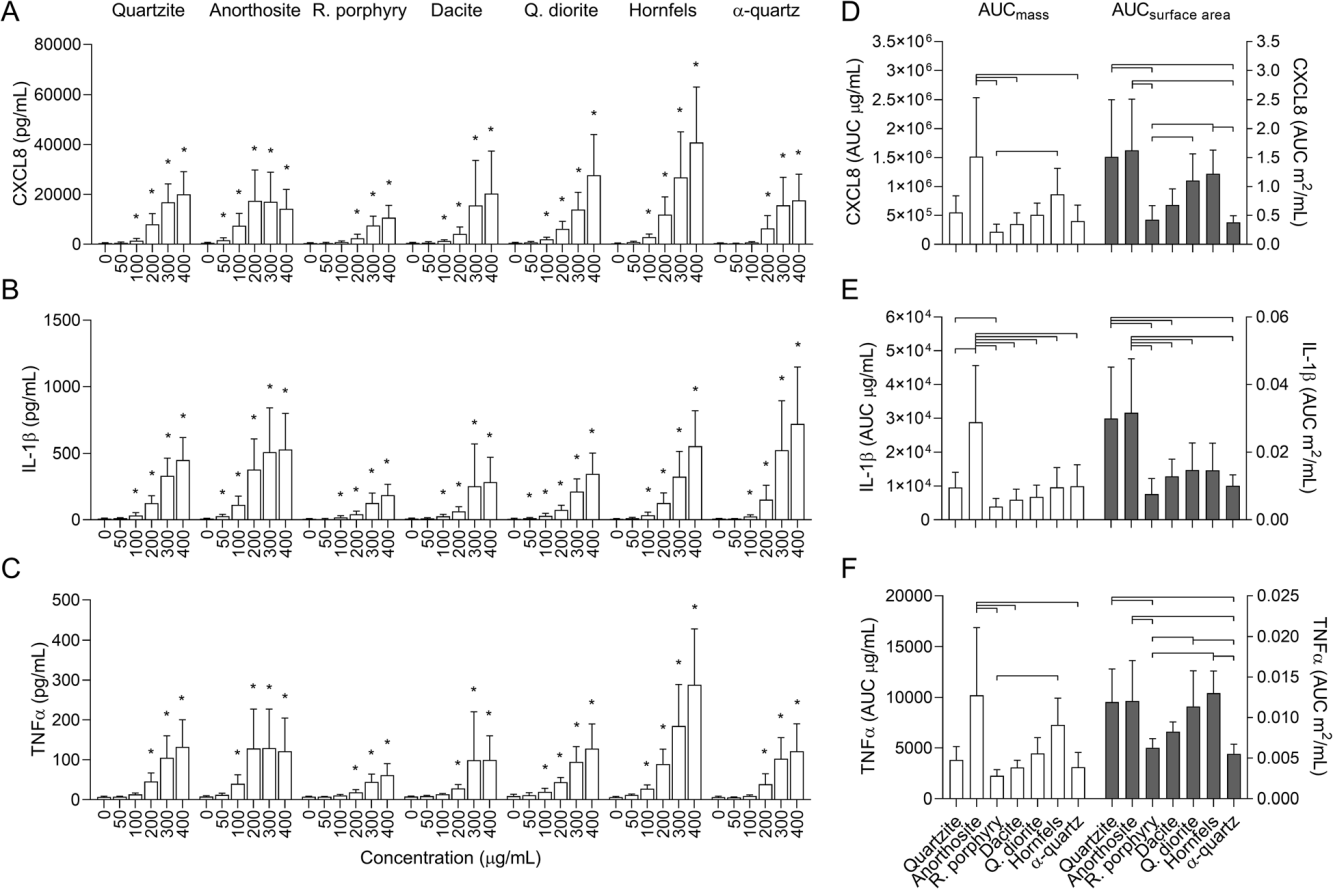
Stone particles induce sample- and concentration-dependent increases of pro-inflammatory cytokines in THP-1 macrophages. THP-1 macrophages were exposed to 0–400 μg/mL quartzite, anorthosite, rhomb porphyry, dacite, quartz diorite, hornfels and α-quartz for 24 h. The release of CXCL8 (**a** and **d**), IL-1β (**b** and **e**) and TNFα (**c** and **f**) in the cell culture supernatant was measured by ELISA. Results are presented as mean ± SD (*n* = 5–6). Area under the curve (AUC) values in (**d**-**f**) were calculated from the values in (**a**-**c**) for the concentration-range of 0–200 μg/mL, and are presented related to equal mass (white bars) and surface area (grey bars). Statistical significance is based on a two-way ANOVA followed by Dunnet’s post-test (**a**-**c**) or a one-way ANOVA followed by Tukey post-test (**d**-**f**). Values were log-transformed prior to statistical analysis based on deviation from normality and homoscedasticity. Asterisks (*) indicate statistically significant difference from the respective control (**a**-**c**), while a capped line indicates statistically significant difference between samples (**d**-**f**)

All particle samples induced concentration-dependent increases in CXCL8, IL-1β and TNFα secretion in THP-1 macrophages, reaching statistical significance at 100 or 200 μg/mL in most cases (Fig. 4a-c). Compared with HBEC3-KT, the particle-induced CXCL8 and IL-1β responses were higher in THP-1 macrophages, with the exception of CXCL8 induced by the α-quartz sample, which was of a similar magnitude in both cell models. In general, the effect of α-quartz was lower relative to the other particle samples in THP-1 than in HBEC3-KT cells, possibly reflecting the lower amount of cytokine-binding detected in the RPMI medium compared with DMEM. As for HBEC3-KT, the concentration range of 0–200 μg/mL was chosen for between particle-comparison of AUC values due to cytotoxicity at the highest concentrations (Fig. 2b and e). At an equal mass basis, anorthosite induced the highest cytokine responses, which were significantly higher than all the other particle samples for IL-1β, and higher than rhomb porphyry, dacite and α-quartz for CXCL8 and TNFα (Fig. 4d-f). Rhomb porphyry was consistently the least potent particle sample in our tests, although most of the differences did not reach statistical significance (Fig. 4d-f). As with HBEC3-KT cells, adjusting the AUC values for differences in surface area altered the order of potency between samples. Compared with the responses on an equal mass basis, the effect of quartzite was increased relative to the other particle samples, while the effect of anorthosite was diminished. At equal surface area concentrations, the quartzite, anorthosite, quartz diorite and hornfels samples induced similar levels of CXCL8 and TNFα, while rhomb porphyry and α-quartz were the least potent. The differences were statistically significant in all cases except between quartz diorite and α-quartz for CXCL8 (Fig. 4d and f). For IL-1β, quartzite and anorthosite were the most potent samples and induced similar responses that were significantly higher than rhomb porphyry, dacite and α-quartz. In addition, anorthosite induced significantly higher levels of IL-1β than quartz diorite (Fig. 4e).

**Fig. 5:**
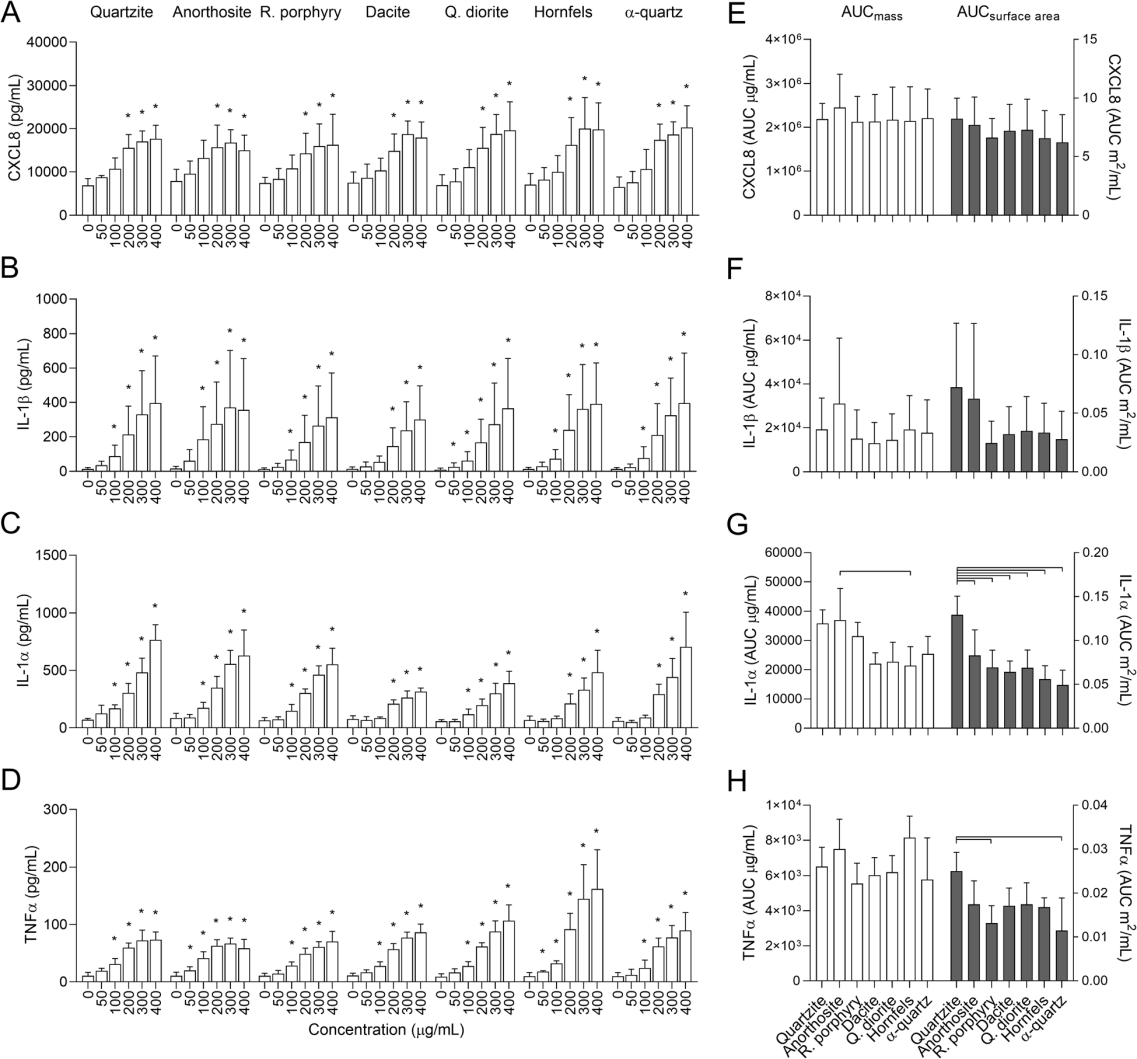
Stone particles induce sample- and concentration-dependent increases of pro-inflammatory cytokines in a HBEC3-KT/THP-1 co-culture. Co-cultures of HBEC3-KT cells and THP-1 macrophages were exposed to 0–400 μg/mL quartzite, anorthosite, rhomb porphyry, dacite, quartz diorite, hornfels and α-quartz for 24 h. The release of CXCL8 (**a** and **e**), IL-1β (**b** and **f**), IL-1α (**c** and **g**) and TNFα (**d** and **h**) in the cell culture supernatant was measured by ELISA. Results are presented as mean ± SD (*n* = 4). Area under the curve (AUC) values in (**e**-**h**) were calculated from the values in (**a**-**d**) for the concentration-range of 0–200 μg/mL, and are presented related to equal mass (white bars) and surface area (grey bars). Statistical significance is based on a two-way ANOVA followed by Dunnet’s post-test (**a**-**d**) or a one-way ANOVA followed by Tukey post-test (**e**-**h**). Values in (**a**-**d**) were log-transformed prior to statistical analysis based on deviation from normality and homoscedasticity. Asterisks (*) indicate statistically significant difference from the respective control (**a**-**d**), while a capped line indicates statistically significant difference between samples (**e**-**h**)

Similar to the corresponding monocultures, all particle samples induced concentration-dependent increases in CXCL8, IL-1β, IL-1α and TNFα in the co-culture of HBEC3-KT cells and THP-1 macrophages (Fig. 5a-d). Particle-induced CXCL8 and IL-1β cytokine responses were generally higher in the co-culture than the HBEC3-KT cells, while the levels were similar between the co-culture and THP-1 macrophages. Moreover, the basal CXCL8 secretion was greater in the co-culture than in the HBEC3-KT cells and THP-1 macrophages alone. Compared to the monocultures, the differences between particle samples were less evident in the co-culture. Apart from the higher levels of IL-1α induced by anorthosite compared to hornfels, no statistically significant differences between the samples were observed for any of the cytokines at an equal mass basis (Fig. 5e-h). At equal surface area concentrations, quartzite induced significantly higher IL-1α levels than all the other particle samples and a higher TNFα response than rhomb porphyry and α-quartz (Fig. 5g and h). No significant differences were detected between any of the particle samples for CXCL8 and IL-1β release at either dose metric (Fig. 5e and f).

The contribution of the individual cell types in the co-culture responses was determined using conditioned medium from HBEC3-KT and THP-1 monocultures cultured and exposed to α-quartz under the same conditions as in the co-culture. The results suggest that HBEC3-KT cells secreted higher amounts of CXCL8 than the THP-1 macrophages, and that transfer of conditioned medium from HBEC3-KT cells to THP-1 macrophages induced an additional increase in CXCL8 that was close to the level in the co-culture (Figure S2A). Thus, HBEC3-KT cells appeared to be responsible for the majority of the effect on CXCL8 secretion in the co-culture, either directly due to particle exposure or through activation of the THP-1 macrophages. However, the transfer of conditioned media failed to replicate the increased CXCL8 basal levels observed in the co-culture (Figure S2A). The IL-1β response in the co-culture was higher than both HBEC3-KT cells and THP-1 macrophages, which released similar levels (Figure S2B). Moreover, the transfer of conditioned media between the monocultures failed to replicate the IL-1β response in the co-culture, suggesting that the additional effect in the co-culture requires the cells to be grown together (Figure S2B). The IL-1α response in HBEC3-KT cells was similar to the co-culture at 200 μg/mL, while THP-1 macrophages secreted very low levels that were below the limit of detection for the assay, suggesting that the HBEC3-KT cells are responsible for the majority of the IL-1α secretion in the co-culture (Figure S2C). However, although conditioned medium from THP-1 macrophages only induced very low levels of IL-1α in the HBEC3-KT cells, transfer of medium from particle-exposed HBEC3-KT cells to THP-1 macrophages caused an additional increase in IL-1α secretion that was similar to the co-culture at 400 μg/mL, suggesting that the THP-1 macrophages may also contribute to the IL-1α response (Figure S2C). The THP-1 macrophages secreted the highest levels of TNFα, while only a small response was detected after exposing the HBEC3-KT cells (Figure S2D). Transfer of HBEC3-KT medium to THP-1 macrophages induced a robust increase in TNFα that was similar to the response in the co-culture (Figure S2D). Thus, the THP-1 macrophages appeared to be responsible for the majority of the TNFα response in the co-culture, either due to direct effects of particle exposure or in response to particle-induced mediators from HBEC3-KT cells.

### Hemolysis

**Fig. 6:**
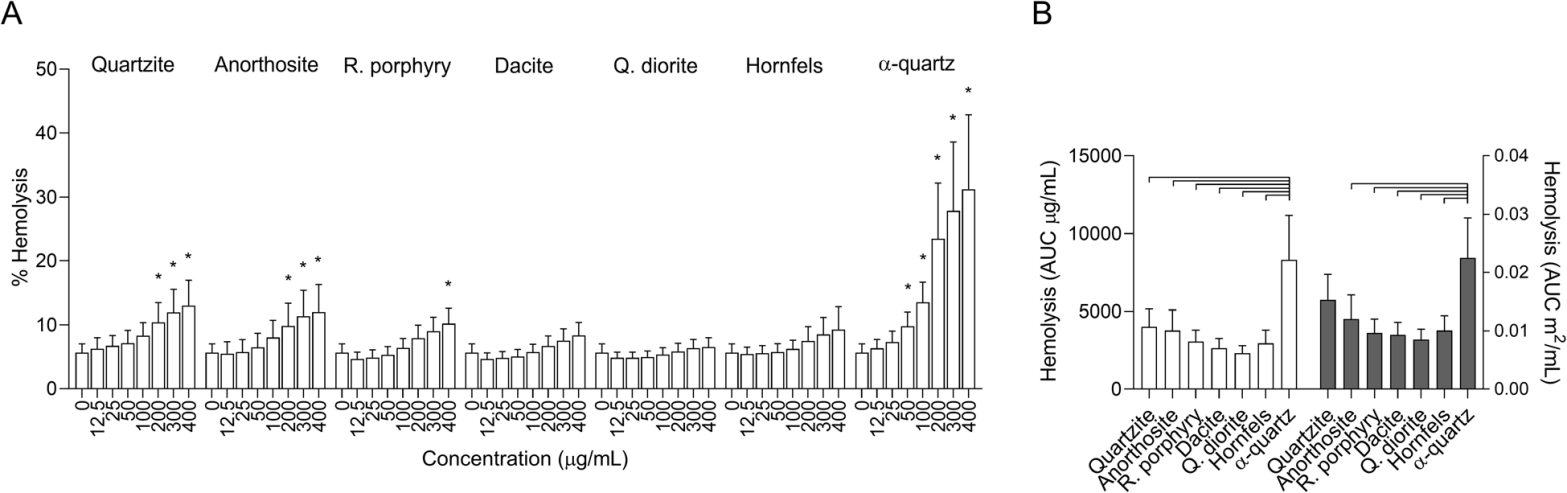
Stone particles induce sample- and concentration-dependent lysis of human erythrocytes. Human erythrocytes harvested from donors were exposed to 0–400 μg/mL quartzite, anorthosite, rhomb porphyry, dacite, quartz diorite, hornfels and α-quartz for 30 min. Free hemoglobin in the supernatant, an indicator of cell lysis, was detected by reading absorbance at 540 nm. Results are presented as mean ± SD (*n* = 4). Area under the curve (AUC) values in (**b**) were calculated from the values in (**a**) for the concentration-range of 0–400 μg/mL, and are presented related to equal mass (white bars) and surface area (grey bars). Statistical significance is based on a two-way ANOVA followed by Dunnet’s post-test (**a**) or a one-way ANOVA followed by Tukey post-test (**b**). Values in (**a**) were log-transformed prior to statistical analysis to satisfy model assumptions of normality and homoscedasticity. Asterisks (*) indicate statistically significant difference from the respective control (**a**), while a capped line indicates statistically significant difference between samples (**b**)

The toxicity of crystalline silica has been linked to interactions between the reactive surface of the particles and lysosomal membranes [36, 37]. To determine if this was also the case for other minerals, the ability of the stone particle samples to lyse biological membranes was assessed using human erythrocytes. Particle-induced hemolysis has been used as a model for lysosomal membrane rupture and has been shown to predict the toxicity of crystalline silica particles [27, 35]. Accordingly, α-quartz had the highest hemolytic activity of the particle samples and induced approximately 30% lysis of erythrocytes at the highest concentration, and a significant increase from control values at 50 μg/mL (Fig. 6a). Both the quartzite and anorthosite samples induced a significant increase in hemolysis at 200 μg/mL and approximately 10% hemolysis at the highest concentration (Fig. 6a). Rhomb porphyry induced a small significant increase at the highest concentration of 400 μg/mL, while no statistically significant difference in hemolysis was detected for dacite, quartz diorite and hornfels at any of the concentrations tested (Fig. 6a). When comparing AUC values calculated from the whole concentration-range of 0–400 μg/mL, α-quartz induced significantly more hemolysis than all the other particle samples (Fig. 6b). As described for the cytotoxicity and cytokine release data, AUC values were adjusted for differences in surface areas by changing the dose metric to m^2^/mL and fitting a curve to the data before estimating new AUC values from the curve in the concentration-range that was common to all the samples. Comparable concentration-ranges for quartzite, anorthosite, rhomb porphyry, dacite, quartz diorite, hornfels and α-quartz when adjusted for surface area were 0–400, 0–211, 0–211, 0–292, 0–298, 0–253 and 0–217 μg/mL, respectably. At equal surface area, α-quartz induced significantly more hemolysis than all samples except quartzite (Fig. 6b).

### Associations between cellular endpoints

The associations between cellular endpoints were assessed by linear regression, using the mean AUC value for each particle sample. Using AUC values calculated for the full concentration-range of 0–400 μg/mL at an equal mass basis, significant associations were detected between reductions in cell viability and increased levels of CXCL8 (β = − 29,924, R^2^ = 0.75, *p* < 0.05), IL-1β (β = − 450, *R*^2^ = 0.93, *p* < 0.001) and IL-1α (β = − 1821, *R*^2^ = 0.91, *p* < 0.001) in HBEC3-KT (Figure S3). Significant associations between reduced cell viability and increased secretion of IL-1β (β = − 1292, *R*^2^ = 0.67, *p* < 0.05) and IL-1α (β = − 2373, *R*^2^ = 0.60, *p* < 0.05) were also detected in the co-culture of HBEC3-KT and THP-1 macrophages (Figure S4), although the fit of the model was lower than for the HBEC3-KT monoculture as evident by the lower R^2^ values. In contrast, no significant associations between viability and cytokine release were detected in THP-1 macrophages (Figure S5). When adjusting AUC values to reflect exposure at the same surface area concentrations, the significant associations remained between reductions in cell viability and increases in CXCL8 (β = − 22,039, *R*^2^ = 0.64, *p* < 0.05), IL-1β (β = − 507, *R*^2^ = 0.88, *p* < 0.01) and IL-1α (β = − 1285, *R*^2^ = 0.85, *p* < 0.01) in HBEC3-KT (Figure S3) and with IL-1β (β = − 1244, *R*^2^ = 0.72, *p* < 0.05) and IL-1α (β = − 2138, *R*^2^ = 0.83, *p* < 0.01) in the HBEC3-KT and THP-1 co-culture (Figure S4). In addition, a significant association was detected between reduced cell viability and increases in IL-1β (β = − 800, *R*^2^ = 0.83, *p* < 0.01) in THP-1 macrophages after adjusting for the surface areas of the particles (Figure S5).

Significant associations were detected between release of CXCL8 (β = 80, *R*^2^ = 0.96, *p* < 0.001), IL-1β (β = 1.2, *R*^2^ = 0.86, *p* < 0.01) and IL-1α (β = 2.6, *R*^2^ = 0.69, *p* < 0.05) in HBEC3-KT cells and particle-induced hemolysis (Figure S6). However, the relationship was highly dependent on the α-quartz reference sample and removing this data point made the associations non-significant (Data not shown). When adjusting the AUC values for differences in surface area, no associations were detected between cytokine release and hemolysis in HBEC3-KT cells (Figure S6). Moreover, no significant associations were detected between cytokine release and hemolysis in THP-1 macrophages or the co-culture of HBEC3-KT and THP-1 cells at either dose metric (Data not shown).

### The role of the NLRP3 inflammasome

**Fig. 7:**
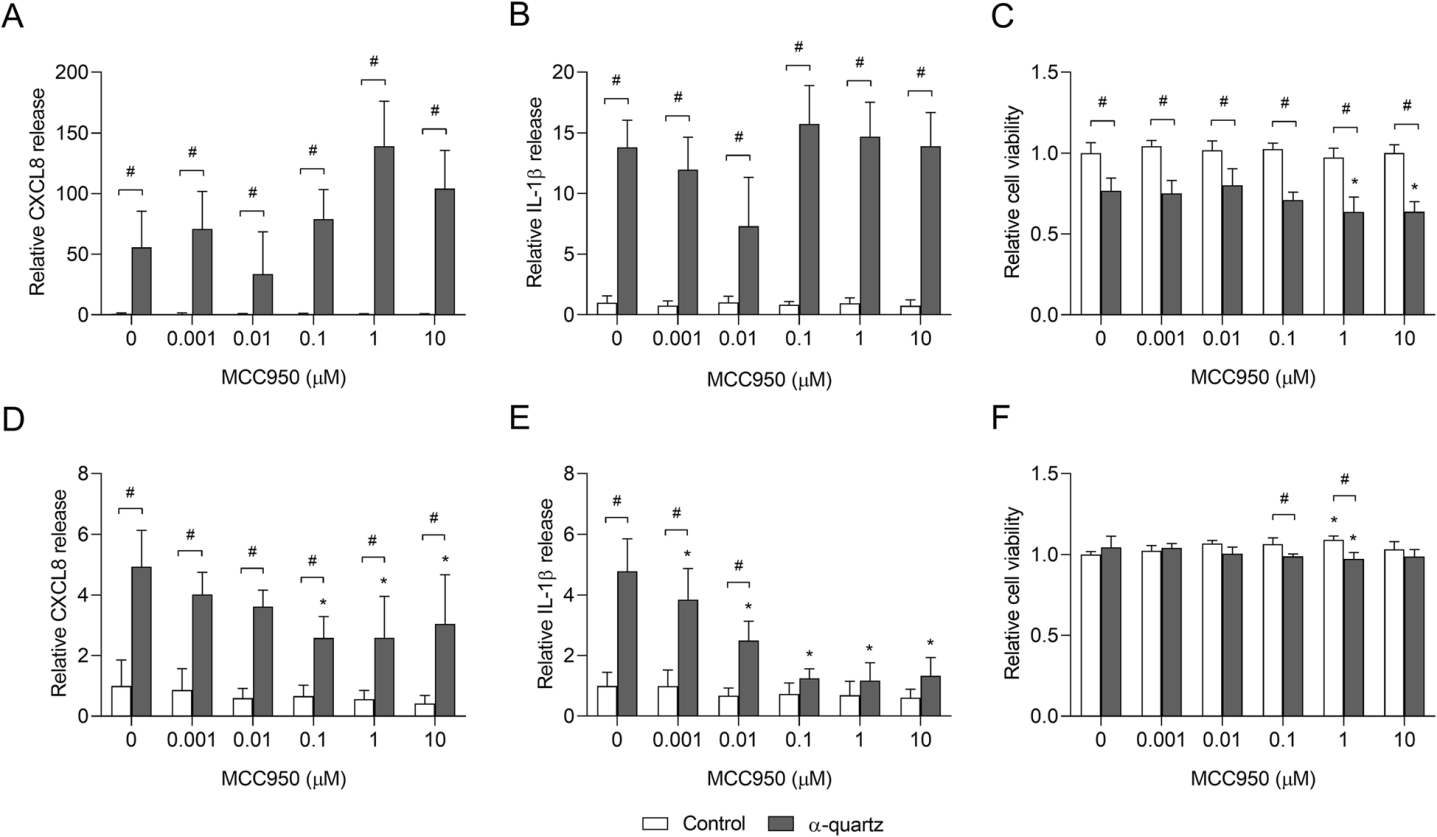
The effect of MCC950 on α-quartz-induced cytokine release and cytotoxicity in HBEC3-KT cells and THP-1 macrophages. HBEC3-KT cells (**a**-**c**) and THP-1 macrophages (**d**-**f**) were exposed to 0–10 μM MCC950 and incubated for 30 min before adding 200 μg/mL α-quartz and incubating the cells for 24 h. The levels of CXCL8 (**a** and **d**) and IL-1β (**b** and **e**) in the cell culture supernatant were detected by ELISA while the cell viability (**c** and **f**) was determined by alamarBlue© assay. Results are presented as mean ± SD (*n* = 5) and are normalized to the mean response of each experiment. Statistical significance is based on a two-way ANOVA followed by Dunnet’s and Sidak post-tests. Values in (**a** and **b**) were log-transformed prior to statistical analysis to satisfy model assumptions of normality and homoscedasticity. * Statistically significant difference from the respective control. # Statistical significance between exposure groups

Activation of the NLRP3 inflammasome is involved in the adverse effects of several particulate compounds, causing maturation and release of IL-1β. Thus, the potential role of NLRP3 in mediating the stone particle-induced effects was assessed using the specific inhibitor MCC950 [54]. As quartz is a known NLRP3 activator, the ability of the MCC950 to block NLRP3-mediated responses in HBEC3-KT cells and THP-1 macrophages was tested with the α-quartz reference sample (Fig. 7). MCC950 was not cytotoxic by itself and did not induce cytokine responses at any of the concentrations tested, in neither HBEC3-KT cells nor THP-1 macrophages (Fig. 7). However, combined exposure with α-quartz at higher concentrations caused a slight decrease in cell viability in both cell models at the highest concentrations (Fig. 7c and f). Pre-incubation with 0.001–10 μM MCC950 did not cause any statistically significant effect on α-quartz-induced CXCL8 and IL-1β responses in HBEC3-KT cells (Fig. 7a and b). However, a small but consistent decrease in both cytokines was observed at 0.01 μM. In contrast, MCC950 caused a marked and concentration-dependent decrease in α-quartz-induced CXCL8 and IL-1β release in THP-1 macrophages that reached maximum inhibition at 0.1 μM (Fig. 7d and e). The effect was greatest on IL-1β secretion, reducing the particle-induced response almost to control levels (Fig. 7e).

**Fig. 8:**
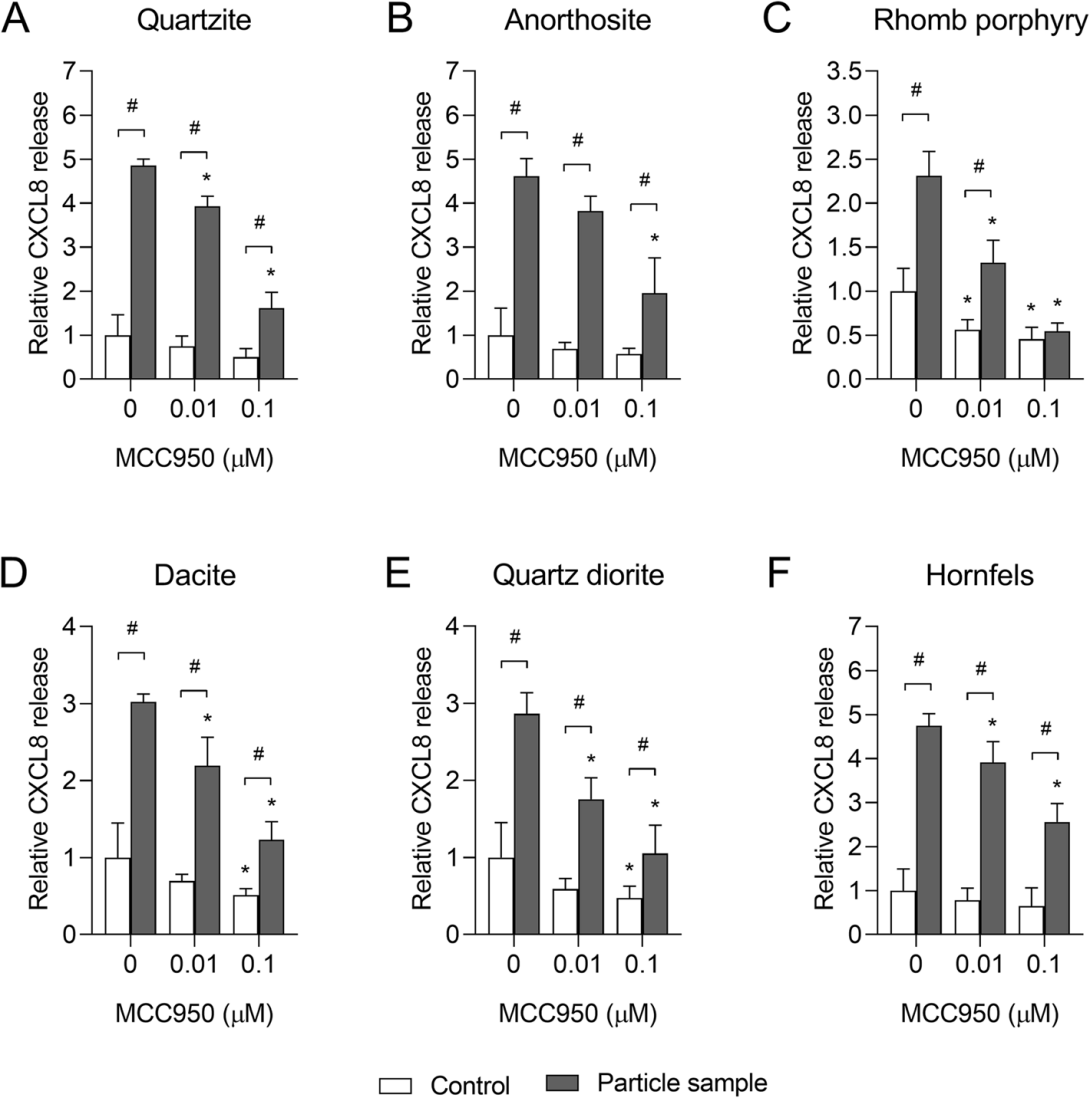
MCC950 induces a concentration-dependent decrease in stone particle-induced CXCL8 release in THP-1 macrophages. THP-1 macrophages were exposed to 0–0.1 μM MCC950 and incubated for 30 min before adding 200 μg/mL quartzite (**a**), anorthosite (**b**), rhomb porphyry (**c**), dacite (**d**), quartz diorite (**e**) and hornfels (**f**), and incubating the cells for 24 h. The level of CXCL8 in the cell culture supernatant was detected by ELISA. Results are presented as mean ± SD (*n* = 4) and are normalized to the mean response of each experiment. Statistical significance is based on a two-way ANOVA followed by Dunnet’s and Sidak post-tests. * Statistically significant difference from the respective control. # Statistically significant difference between exposure groups

**Fig. 9:**
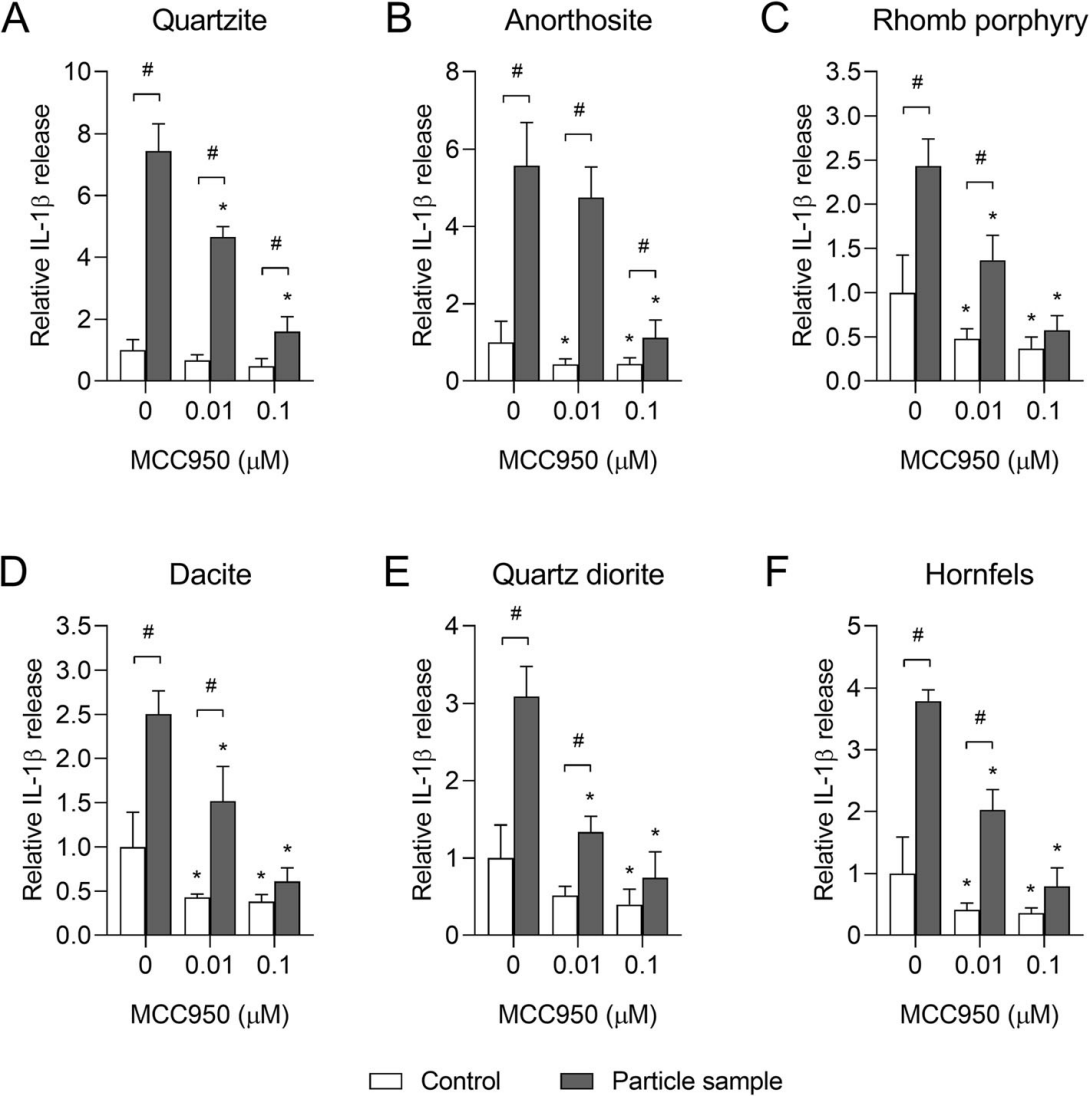
MCC950 induces a concentration-dependent decrease in stone particle-induced IL-1β release in THP-1 macrophages. THP-1 macrophages were exposed to 0–0.1 μM MCC950 and incubated for 30 min before adding 200 μg/mL quartzite (**a**), anorthosite (**b**), rhomb porphyry (**c**), dacite (**d**), quartz diorite (**e**) and hornfels (**f**), and incubating the cells for 24 h. The levels of IL-1β in the cell culture supernatant was detected by ELISA. Results are presented as mean ± SD (*n* = 4) and are normalized to the mean response of each experiment. Statistical significance is based on a two-way ANOVA followed by Dunnet’s and Sidak post-tests. Values in **b** were log-transformed prior to statistical analysis to satisfy model assumptions of normality and homoscedasticity. * Statistically significant difference from the respective control. # Statistical significance between exposure groups

**Fig. 10:**
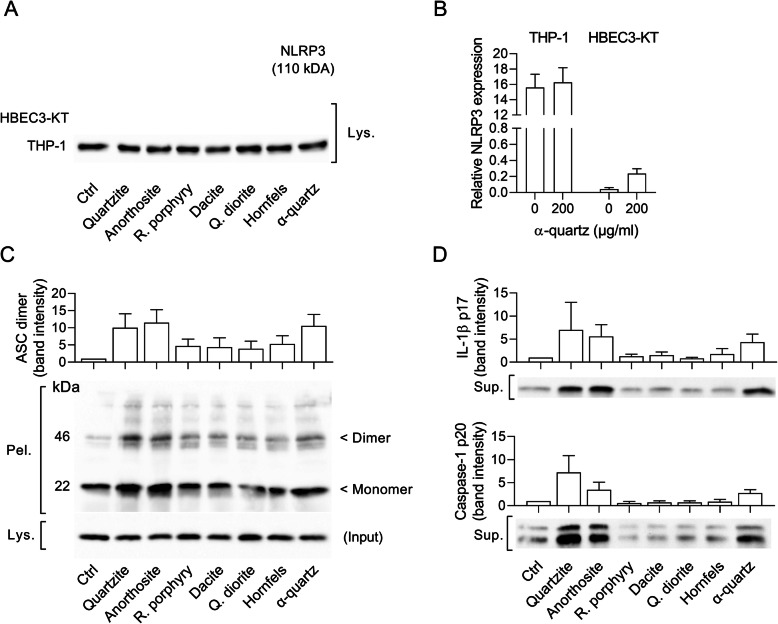
Stone particles activate the NLRP3 inflammasome in THP-1 macrophages. HBEC3-KT cells and THP-1 macrophages were exposed to 200 μg/mL quartzite, anorthosite, rhomb porphyry, dacite, quartz diorite, hornfels and α-quartz for 12 h. Western blot analysis was used to determine the levels of NLRP3 and ASC in the cell lysate (**a** and **c**), ASC oligomers in cross-linked pellets (**c**), and cleaved caspase-1 and IL-1β in the cell culture supernatant (**d**). NLRP3 expression in cell lysates was also measured using ELISA (**b**). The results of the western blot analyses are presented as representative blots of cell culture supernatant (Sup), cell lysates (Lys) and insoluble pellets (Pel), and the mean ± SD relative band intensity of three independent experiments. The band intensity values in **c** has been normalized to the ASC expression in the cell lysates (Input). ELISA results are presented as mean ± SD (*n* = 3) and are normalized to the total protein concentration in each sample

Based on these results, 0.01 and 0.1 μM MCC950 were chosen for experiments with the stone particle samples. Pre-incubation of THP-1 macrophages with 0.01 and 0.1 μM MCC950 significantly decreased particle-induced CXCL8 and IL-1β for all samples, suggesting that the stone particle-induced responses are dependent on NLRP3 in this model system (Figs. 8 and 9). However, as observed for α-quartz, MCC950 did not cause any statistically significant reductions in the stone particle-induced cytokine responses in HBEC3-KT cells (Data not shown). In line with the lack of effect of MCC950, only very low levels of NLRP3 could be detected in the HBEC3-KT cells, although a small increase was observed after 12 h exposure to 200 μg/mL α-quartz (Fig. 10a and b). Conversely, THP-1 macrophages expressed high amounts of NLRP3 protein in control and particle-exposed cells (Fig. 10a and b). To confirm that the inflammasome was activated in the THP-1 macrophages, the formation of ASC oligomers and cleaved IL-1β and caspase-1 was assessed by western blot after 12 h exposure to 200 μg/mL stone particles. Compared with unexposed cells, increases in ASC monomers and dimers were detected in THP-1 macrophages after 12 h exposure, suggesting that exposure to the stone particles leads to activation of the inflammasome complex (Fig. 10c). Moreover, an increase in caspase-1 p20 and IL-1β p17 was also detected, further suggesting that the effects of the stone particles are mediated through activation of NLRP3 in the THP-1 macrophages (Fig. 10d). However, no increase in ASC oligomers or cleaved caspase-1 and IL-1β was detected in HBEC3-KT cells after exposure to the stone particle samples (Data not shown), suggesting that the responses are mediated through another mechanism in this cell model.

## Discussion

Respirable mineral dusts represent a potential health hazard in industries where rocks and minerals are handled, and may also make up a large portion of ambient PM, a source of exposure for the general population. While the health effects of quartz and asbestos have received considerable attention in the literature, much less is known about the potential effects of other mineral particles. In the present study, the cytotoxic, pro-inflammatory and membranolytic effects of six stone particle samples of different mineral composition were assessed in cell culture models representing the human airways. Moreover, the role of the NLRP3 inflammasome in mediating the observed effects was assessed using the specific NLRP3 inhibitor MCC950 and detection of ASC oligomers and the cleaved subunits of caspase-1 and IL-1β. Overall, the results indicate that the ability to induce cytotoxicity and release of pro-inflammatory cytokines in bronchial epithelial cells and macrophage differs between stone particle samples, with some samples inducing similar or higher levels than the α-quartz reference sample. Moreover, the results suggest that the particles exert their effects through different mechanisms depending on the cell type.

At an equal mass basis, the α-quartz reference sample was the most potent in HBEC3-KT cells, both in terms of cytotoxicity and cytokine secretion, followed by quartzite and anorthosite. In THP-1 macrophages, quartzite and anorthosite were the most cytotoxic particle samples, while anorthosite and hornfels induced the highest levels of cytokines. Rhomb porphyry was consistently among the least potent samples in both model systems. While similar trends were observed, few of the apparent differences reached statistical significance in the combined culture of HBEC3-KT cells and THP-1 macrophages, compared with the corresponding monocultures. In line with previous studies from our group, differences in surface area could not fully explain the differences between the particle samples [47, 48], suggesting that the differences in potency were due to other parameters, such as particle composition or surface reactivity. After correcting for differences in surface area, quartzite and anorthosite were the most cytotoxic particle samples in both HBEC3-KT cells and THP-1 macrophages, surpassing the effects of the α-quartz reference sample. Quartzite was the most potent inducer of cytokines in HBEC3-KT cells, while the effect of anorthosite was diminished relative to the other samples. In THP-1 macrophages, quartzite, anorthosite, quartz diorite and hornfels were the most potent samples in terms of CXCL8 and TNFα release, while quartzite and anorthosite induced the highest levels of IL-1β.

In addition to the qualitative differences in the order of potency between the stone particle samples, quantitative differences in cytokine release were observed in the different cell models. The particle-induced CXCL8 and IL-1β responses were lower in the HBEC3-KT cells compared to the THP-1 macrophages and the co-culture model, which released cytokine levels of a similar magnitude. In addition to the differences in particle-induced responses, larger basal cytokine levels were detected in the co-culture compared to the HBEC3-KT and THP-1 monocultures. Previous studies have reported larger effects from particle exposure in co-cultures of macrophages and epithelial cells than in the corresponding monocultures [55–57]. Moreover, an increase in basal responses of TNFα and MIP-2 has been reported in a co-culture model of rat macrophages and epithelial cells exposed to particles [55]. Jimenez et al. reported that medium from macrophages exposed to ambient PM_10_ induces expression of CXCL8 in A549 epithelial cells through TNFα-mediated NFκB activation [58], suggesting that secreted mediators may account for the differences between the monocultures and the co-culture. Moreover, recent studies from our group suggest that particle-induced responses in a complex 3D culture of macrophages, epithelial cells and endothelial cells depend on activation of IL-1R1, indicating a role for IL-1α and/or IL-1β [59]. In the present study, transfer of conditioned medium suggested that the HBEC3-KT cells were responsible for the majority of the CXCL8 response in the co-culture, either directly due to particle exposure or through activation of THP-1 macrophages via HBEC3-KT-derived mediators. Moreover, particle-induced mediators from the HBEC3-KT cells induced TNFα secretion in THP-1 macrophages, further indicating a central role for the HBEC3-KT cells. However, the transfer of conditioned media failed to replicate the increased IL-1β response and the high basal CXCL8 secretion observed in the co-culture, suggesting that growing HBEC3-KT cells and THP-1 macrophages together results in responses that cannot be fully predicted by the activity of the individual monocultures.

Non-specific binding of cytokines to particles has been reported in previous studies and may vary between particle samples of different composition, possibly confounding the results of bioassays such as ELISA [53]. In the present study, the differences in avidity between the particle samples were negligible in the RPMI medium used with THP-1 macrophages, but of greater concern in the DMEM medium used with HBEC3-KT cells. The binding was particularly prominent for CXCL8 and IL-1β, but practically non-existent for IL-1α. However, there was good agreement between the results for these cytokines in the cell culture experiments in regards to the potency of the stone particle samples, suggesting that binding has not overly affected the results. Preliminary results of particle-induced gene expression of CXCL8 and IL-1β suggest that the primary effect of non-specific binding is the differences in potency between the stone particle samples and the α-quartz reference sample (Figure S7). Thus, the relative potency of the stone particle samples compared to quartz may have been underestimated in HBEC3-KT cells. Moreover, the effect of anorthosite on CXCL8 release may have been somewhat underestimated compared to the other stone particle samples, as it bound the most CXCL8 of all the tested samples.

The high potency of the anorthosite and hornfels samples contradicts previous studies of stone- and mineral particles indicating that particle samples with high feldspar content have low cytotoxic and pro-inflammatory activity [45–48, 50]. In the present study, the anorthosite, hornfels and rhomb porphyry samples all consisted primarily of feldspar minerals, but varied considerably in potency, suggesting that the total content of feldspar minerals is a poor indicator of stone-particle toxicity. However, as the samples contain different feldspar minerals, in the form of K-feldspar and plagioclase feldspar, as well as other minerals such as muscovite, hornblende, chlorite, calcite, epidote and quartz, differences in mineral composition may still be a possible explanation for the differences in potency. Thus, the results of the present study indicate that mineral particles other than quartz and asbestos may warrant more attention in future research.

In line with the known toxicity of quartz, particle samples consisting primarily of quartz, such as quartzite and the α-quartz reference sample, were among the most potent in the present study, although the effects relative to the other samples varied between cell models and whether the responses were adjusted for differences in surface area or not. Unexpectedly, the anorthosite sample, which contains no quartz, was equal to or higher in potency than quartzite and α-quartz, while some samples with moderate quartz content, such as dacite and quartz diorite, were among the least potent. This further exemplifies the importance of other particle constituents or properties and suggests that the total quartz content does not fully predict stone particle toxicity. Several experimental studies report that silica particles of different origin vary in toxicity in vivo and in vitro [27, 35, 60, 61], suggesting that different properties of the quartz present in samples may partly explain the differences in cellular responses to the quartz-rich samples quartzite and α-quartz. The comparably low toxicity of quartz diorite and dacite may also be a question about the available quartz concentration, which may not be high enough in mixed dust samples to exceed the concentration needed to elicit toxic responses. When comparing the quartz concentration in each sample, 400 μg/mL of quartz diorite and dacite would be approximately equal to 100 μg/mL of quartzite and α-quartz. At this concentration, the effects of quartzite and α-quartz were lower than the mixed dust samples in all cases. Studies also suggest that contamination of quartz particles with coal mine dust and aluminum-rich clay minerals, as well as aluminum compounds and iron, may decrease the toxicity of the particles [62–64]. As the samples of dacite and quartz diorite consist of a mixture of different minerals in addition to quartz, such as feldspars, muscovite, epidote and chlorite, exposure to quartz dust in a mixture with other minerals could possibly reduce the toxicity of the particle samples.

Several crystalline and particulate compounds have been reported to activate the NLRP3 inflammasome [28–32]. However, to our knowledge this is the first study to report the potential involvement of NLRP3 in effects from mineral particles other than silica or asbestos. In the present study, pre-incubation with the NLRP3-specific inhibitor MCC950 [54] reduced the stone particle-induced secretion of CXCL8 and IL-1β in THP-1 macrophages, suggesting that the inflammatory responses to these particles involve NLRP3 inflammasome activation in this model system. Since CXCL8 is not directly dependent on NLRP3 or caspase-1 for bioactivity and release, the reduction in particle-induced CXCL8 suggests that the CXCL8-response in THP-1 macrophages was partly driven by increased IL-1β secretion. The involvement of the NLRP3 inflammasome was confirmed using western blotting, which showed that exposure to the stone particle samples caused the formation of ASC oligomers and cleavage of pro-caspase-1 and pro-IL-1β. Previous studies suggest that the toxicity of crystalline silica is caused by the reactive surface of the particles, which causes activation of the NLRP3 inflammasome through lysosomal destabilization [30, 34–37]. Thus, a critical question is whether the stone particles examined in the present study activate NLRP3 via a similar mechanism. Human erythrocytes have been used as a model system for membranolysis and lysosomal rupture, and the ability to induce hemolysis has been reported to predict the toxicity of silica particles [27, 35]. In line with this, the α-quartz reference sample induced a substantial amount of hemolysis in the present study. In contrast, the stone particle samples only induced low to no hemolysis at the same concentrations. Moreover, no association was detected between stone particle-induced cytotoxicity or cytokine release in THP-1 macrophages and hemolytic potential. Thus, the results suggest that the stone particle samples activated NLRP3 through a different mechanism than quartz, possibly independent of lysosomal rupture. Alternatively, the results may indicate that the hemolysis assay is not predictive of lysosomal rupture induced by stone particles.

Although inflammasome- or caspase 1-dependent inflammatory responses have been reported in human lung epithelial cells exposed to crystalline silica, ambient particulate matter and nanoparticles [65–67], the NLRP3-specific inhibitor MCC950 had no effect on stone particle-induced cytotoxicity or cytokine release in HBEC3-KT cells in the present study. Moreover, NLRP3 expression was very low and no increase in ASC oligomerisation or cleavage of the pro-forms of caspase-1 and IL-1β was detected, suggesting that the observed effects are mediated through other pathways in this cell model. Linear regression analyses detected significant associations between decreased cell viability and increased cytokine release, which may indicate that particle-induced cytotoxicity promotes the inflammatory response in the HBEC3-KT cells, or that the cytotoxicity and pro-inflammatory responses are orchestrated by common upstream signalling events. In the present study, a concentration-dependent increase in IL-1α was detected in the supernatant after exposure to all stone particle samples in HBEC3-KT. Likewise, IL-1α release has also been reported in lung epithelial cells exposed to carbon black nanoparticles and ambient particulate matter [68, 69]. IL-1α is released during necrotic cell death and may bind to IL-1R1, initiating the transcription of pro-inflammatory genes in the recipient cell [43, 70, 71]. Several studies report that the release of IL-1α, and the subsequent activation of inflammation via IL-1RI, is central to the inflammatory response to tissue damage and necrosis [72–75]. Moreover, IL-1α has been reported to induce CXCL8 in an autocrine manner in A549 epithelial cells infected with respiratory syncytial virus [76]. Thus, IL-1α released through particle-induced cell death could possibly explain the inflammatory response observed in HBEC3-KT in the present study. However, it should be noted that cytokine release seems to occur at lower particle concentrations than cytotoxicity in the HBEC3-KT cells, suggesting that cytotoxicity is not necessarily an upstream event of the cytokine responses. In line with this, previous studies have also reported IL-1α secretion in absence of cytotoxicity in epithelial cells [68, 69, 77]. In addition to IL-1α, the release of other factors associated with cell injury and necrosis, such as high mobility group box 1 (HBMG1), IL-33 and adenosine triphosphate (ATP), has also been reported following particle exposure [39, 67, 78]. As the release of these mediators was not measured in the present study, their involvement in stone particle-induced inflammation cannot be excluded.

## Conclusions

The present study indicates that different stone particle samples can induce acute pro-inflammatory responses in human bronchial epithelial cells and macrophages, acting through NLRP3-dependent and -independent mechanisms. Quartzite, anorthosite, hornfels and quartz diorite were among the most potent samples, depending on the cell model, endpoint and concentration-metric. While quartzite consisted primarily of quartz, anorthosite and hornfels consisted primarily of feldspar minerals, as well as other minerals such as muscovite, epidote, calcite and chlorite, suggesting a role for minerals other than quartz. There is currently no evidence to suggest that the stone particles assessed in the present study can induce fibrotic and carcinogenic effects seen for pathogenic mineral particles, such as quartz and asbestos. However, several of the samples induced an acute inflammatory response to a similar or greater extent than the α-quartz reference sample. Given their ubiquitous presence in the environment and sometimes high concentrations in occupational and ambient air pollution, these minerals warrant further attention.

## Materials and methods

### Preparation and characterisation of particle samples

#### Preparation of the particle samples

Samples of quartzite, anorthosite, rhomb porphyry, dacite, quartz diorite and hornfels were delivered by aggregate producers within a specific grain size (8/16 and 0/20 mm) and prepared by The Norwegian Public Roads Administration. The samples were crushed in a Los Angeles test machine and the resulting fine-grained material sieved to collect the portion < 63 μm.

Particle sizes < 10 μm were separated by gravity settling in deionized water at room temperature (20 °C), for a duration determined by solving the Stokes equation for time. The variables used in the stokes equation were standardized for all samples, and used a density of 2.65 for the particle material, assumed an equivalent spherical diameter of 10 μm, and a water density and viscosity determined for 21 °C. After extraction of the < 10 μm fraction by siphon, the remaining material was resuspended and the procedure repeated once to increase the yield. All < 10 μm particles were collected by centrifuging for 65 min at 9500 RPM in a Beckman Coulter Avanti J-26 XP centrifuge with a JA10 rotor. The particle concentrates were dried in a freeze drier.

Min-U-Sil 5®, a high purity sample of crystalline silica, was provided by U.S. Silica Company (MD, USA). According to the manufacturer, this ground silica is at least 98% SiO_2_ and has a size distribution with typically 96% passing 5 μm and a median diameter of 1.6 μm. Min-U-Sil 5® was used as a reference sample and is referred to as “α-quartz” in the main body of the text.

#### Chemical analyses

The geochemical composition of the samples was analysed with a PANalytical Axios sequential wavelength-dispersive X-ray spectrometer operating with a 4 kW Rh-tube. For major element analysis, the sample material was fused to glass beads with Li_2_B_4_O_7_ at 1200 °C. Loss on ignition was determined after 1 h at 1000 °C. The lower limit of quantification is generally 0.01 wt%, whereas the analytical uncertainty is concentration-dependent, but usually better than 5% rel. (2 σ).

#### Mineralogical analyses

Mineralogical analyses were carried out with a Bruker D8 Advance diffractometer (Cu Kα radiation in 3–75° 2θ range). A detailed description of all measurement parameters is given elsewhere [79]. Mineral identification was performed with automatic/manual peak search & match function with Bruker’s Diffraction EVA V3.1 software using Crystallographic Open Database and PDF4 Minerals database from the International Centre of Diffraction Data. Mineral quantification was performed using Rietveld modelling in TOPAS 5 software with an estimated uncertainty of 2 wt%. For verification purposes, the quantified mineral concentrations were re-calculated into element oxides and compared to XRF-data.

#### Particle size and surface area

Specific surface areas of the particles were estimated by the BET formalism using the relative pressure range from 0.05 to 0.3 of the N_2_ isotherms recorded at liquid nitrogen temperatures (77 K) recorded on a BELSORP Mini instrument. Sample activation was carried out overnight at an external pre-treatment unit (BELPREP II vac) at 80 °C under vacuum prior to a short (2 h) pre-treatment at the BELSORP Mini instrument.

The distribution of particle size was determined by analyses on Beckman Coulter LS13320 Laser Particle size analyser in the 0.017–2000 μm measuring range. The sample suspension was disintegrated by adding dispersant agent sodium pyrophosphate (5%), and then sonicated with MSE ultrasonic disintegrator at amplitude 14 for 5 min. Analytical results are presented as Cumulative volume %. Calculation of the results are based on normalisation, and the whole measuring range equals 100% cumulative.

#### Endotoxin contamination

The content of endotoxin in each particle sample was quantified using the Pierce™ Chromogenic Endotoxin Quant Kit (ThermoFisher Scientific, Waltham, MA, USA) according to the manufacturer’s instructions, with minor alterations. As the turbidity of the particles would interfere with the assay, the plate was centrifuged at 290 x g to pellet the particles immediately after the stop solution was added to the reaction mixture. The supernatant (100 μL) was transferred to a new plate and absorbance read at 405 nm. Particle suspensions and endotoxin standard solutions were prepared using HyClone™ Water (Fisher Scientific, Waltham, MA, USA) and Lonza Pyrogen-free Test Tubes (Fisher Scientific, Waltham, MA, USA).

### Cell cultures and exposure

#### THP-1-derived macrophages

THP-1 monocytes (EEAC sigma) were seeded at a density of 5 × 10^5^ cells/mL in 1640 RPMI cell culture medium with L-glutamine (Gibco, Thermo Fischer Scientific, Waltham, MA, USA) supplemented with sodium pyruvate (Sigma-Aldrich, St. Louis, MO, USA), hepes (Sigma-Aldrich, St. Louis, MO, USA), gentamicin (Gibco, Thermo Fischer Scientific, Waltham, MA, USA) and 10% foetal calf serum (FCS; Biochrom, Berlin, Germany), and maintained at 37 °C in a humidified atmosphere containing 5% CO_2_. The cells were passaged every 2–3 days to maintain proper cell density. Prior to the experiments, the cells were seeded on 6-well Corning® Costar® cell culture plates (Merck, Darmstadt, Germany) at a density of 5 × 10^5^ cells/mL in 2 mL cell culture medium. To initiate differentiation into macrophage-like cells, 64 nM phorbol myrisate acetate (PMA; Merck, Darmstadt, Germany) was added to each well followed by 48 h incubation at 37 °C in an atmosphere containing 5% CO_2_. PMA-differentiated THP-1 cells are referred to as THP-1 macrophages in the main body of the text. Prior to exposure, each well was washed once with 1 mL PBS before adding 1 mL of serum-free RPMI and incubating the plate for an additional 24 h.

#### HBEC3-KT cells

Human bronchial epithelial cells (HBEC3-KT) were cultured in LHC-9 medium (Lonza, Basel, Switzerland) in collagen-coated T75 flasks and maintained at 37 °C in a humidified atmosphere containing 5% CO_2_. The cells were passaged twice every week to ensure appropriate culture conditions. For experimental procedures, cells were seeded on collagen-coated 6-well cell culture plates at a concentration of 220,000 cells per well in 1 mL LHC-9 medium. The LHC-9 medium was replaced after 24 h. Then, 24 h prior to exposure, each well was washed once with 1 mL PBS before replacing the medium with 1 mL of serum-free DMEM (Gibco, Thermo Fischer Scientific, Waltham, MA, USA), supplemented with penicillin-streptomycin (Lonza, Basel, Switzerland), ampicillin (New York, NY, USA) and amphotericin B (Sigma, St. Louis, MO, USA).

#### HBEC3-KT/THP-1 co-culture

HBEC3-KT cells were seeded in 6-well cell culture plates in LHC-9 medium at a concentration of 220,000 cells per well and incubated for 48 h at 37 °C in a humidified atmosphere containing 5% CO_2_. THP-1 cells were cultured in a T175 cell culture flask and differentiated with PMA as outlined above. The differentiated THP-1 macrophages were then loosened by accutase (A6964; Sigma Aldrich, Merck, Darmstadt, Germany) treatment and resuspended in equal amounts of serum-free RPMI and DMEM at a concentration of 100,000 cells/mL. Next, the HBEC3-KT cell cultures were washed once with 1 mL PBS before adding 1 mL of THP-1 suspension. The co-cultures were then incubated for 24 h at 37 °C in a humidified atmosphere containing 5% CO_2_. For experiments with conditioned medium, HBEC3-KT and THP-1 cells were cultured and seeded at the same densities and under the same conditions as outlined above. However, THP-1 macrophages were seeded on new 6-well cell culture plates after being loosened with accutase, while the medium of the HBEC3-KT cells was changed to equal amounts of serum-free RPMI and DMEM.

#### Exposure regime and preparation of particle stocks

Particle stocks were prepared in serum-free cell culture medium. To ensure even suspension of the particles, the stock solutions were sonicated for 5 min on ice using a Vibra-Cell™ probe sonicator (Sonics & Materials Inc., Newtown, CT, USA). The primary stock concentration of 2 mg/mL was further diluted in the wells to yield the final concentrations of 50, 100, 200, 300 and 400 μg/mL. HBEC3-KT cells, THP-1 macrophages and HBEC3-KT/THP-1 co-culture were exposed in 1 mL serum-free DMEM, RPMI and DMEM/RPMI, respectively. For experiments assessing the role of NLRP3, cells were incubated with medium containing 0.001, 0.01, 0.1, 1 and 10 μM of the NLRP3 inhibitor MCC950 (Invivogen, San Diego, CA, USA) for 30 min prior to particle exposure. MCC950 was dissolved in dimethyl sulfoxide (DMSO; Sigma-Aldrich, St. Louis, MO, United States), according to the manufacturer’s instructions. The level of DMSO was kept below 0.05% of the total well volume and was equalized between wells. Following exposure, the cells were incubated at 37 °C in a humidified atmosphere containing 5% CO_2_ for 12 or 24 h, depending on the experiment. Next, the cell culture supernatants were transferred to Eppendorf tubes and centrifuged at 290 x g for 10 min to remove cells and debris. The supernatant was then centrifuged for an additional 10 min at 1200 x g to remove particles, before further analyses. For experiments with conditioned medium as described in the "HBEC3-KT/THP-1 co-culture" section, the particle-free supernatants were used to expose HBEC3-KT cells and THP-1 macrophages for 24 h under the same conditions as for particle exposure. Samples from finished experiments were stored at − 80 °C awaiting analysis.

### Cytokine analysis by enzyme-linked immunosorbent assay

The concentrations of cytokines in the cell culture supernatant were measured using Cytoset (TNFα and CXCL8; Invitrogen by Thermo Fischer Scientific, Waltham, MA, USA and Novex by Life Technologies, Waltham, MA, USA) or Duoset (IL-1α and IL-1β; R&D systems, Inc., Minneapolis, MN, USA) ELISA kits, while NLRP3 expression in cell lysates was determined using a SimpleStep ELISA kit (Abcam, Cambridge, United Kingdom). All kits were applied according to the manufacturer’s instructions. ELISA was performed in Nunc Maxisorb plates (Thermo Scientific, Waltham, MA, USA). Absorbance was measured using a Tecan Sunrise plate reader (Tecan, Männedorf, Switzerland).

### Non-specific binding of cytokines to the stone particle samples

Cytokine stocks were prepared in serum-free RPMI and DMEM medium using the standard samples from ELISA kits and incubated with 400 μg/mL quartzite, anorthosite, rhomb porphyry, dacite, quartz diorite, hornfels and α-quartz in 24-well cell culture plates for 24 h at 37 °C in a humidified atmosphere containing 5% CO_2_. Following incubation, the samples were transferred to 1.5 mL Eppendorf tubes and centrifuged for 10 min at 1200 x g to remove particles. The cytokines remaining in the cell culture media was quantified using ELISA as described in the "Cytokine analysis by enzyme-linked immunosorbent assay" section.

### Cytotoxicity

Cytotoxicity was assessed by alamarBlue™ assay (Invitrogen by Thermo Fischer Scientific, Waltham, MA, USA) according to the manufacturer’s instructions. AlamarBlue™ solution was diluted 1:10 in serum-free cell culture medium and added to the cell culture at a volume of 1 mL per well. The cells were then incubated for 60 min at 37 °C in an atmosphere containing 5% CO_2_. Following incubation, 100 μL supernatant was transferred to a 96-well cell culture plate and fluorescence emission measured using a CLARIOstar® plate reader (BMG LABTECH, Ortenberg, Germany).

### Hemolysis assay

The hemolysis assay was adapted from Pavan et al. [34] with some modifications. Fresh donor blood was sampled in a 10 mL BD vacutainer containing K_2_EDTA (BD, Franklin Lakes, NJ, USA) and separated by centrifugation at 1200 x g for 10 min. The serum and buffy coat were then discarded and the erythrocytes resuspended in 0.9% saline. The cells were washed five times and finally resuspended to a concentration of 5% erythrocytes in 0.9% saline. Next, 75 μL cell suspension was added to a 96-well cell culture plate and gently mixed with 150 μL particle solution, or positive (0.1% triton X-100, Thermofischer, Waltham, MA, USA) or negative controls (0.9% saline). The plate was then incubated for 30 min at room temperature on an orbital plate shaker, centrifuged at 290 x g for 5 min, and 75 μL supernatant transferred to a new plate. Absorbance was measured at 540 nm to detect hemoglobin in the supernatant. Results are presented as a percentage of the positive control.

### Immunoblotting to detect ASC oligomers and cleaved IL-1β and caspase-1

#### Western blotting

Detection of ASC oligomers, NLRP3 and cleaved caspase-1 and IL-1β was detected using western blot analysis as described in Låg et al. [80]. Briefly, samples for western blot were suspended in laemmli sample buffer consisting of 10% glycerol (Merck, Darmstadt, Germany) 5% mercaptoethanol (Sigma, St. Louis, MO, USA), 2% sodium dodecyl sulphate (SDS) (Sigma, St. Louis, MO, USA) and 0.01% bromophenol blue (Sigma, St. Louis, MO, USA), and incubated at 95 °C for 10 min to denature the proteins. The proteins were then separated using 8 and 15% SDS-PAGE gel electrophoresis (BioRad, Hercules, California, USA) and transferred to a nitrocellulose membrane (Maine Manufacturing LLC, Sanford, ME, USA). The membranes were incubated overnight with specific antibodies against ASC (Santa Cruz Biotechnology, Dallas, Texas, USA.), IL-1β (Cell Signalling Technology, Danvers, Massachusetts, USA) and caspase-1 (Cell Signalling Technology, Danvers, Massachusetts, USA) 3% milk or in 5% bovine serum albumin (BSA). Next, the membranes were incubated with HRP-conjugated anti-mouse or anti-rabbit secondary antibodies (Dako, Santa Clara, Ca, USA) and SuperSignal West Dura (Thermo scientific, Waltham, MA, USA). Chemiluminescence was monitored and recorded using a Chemi-Doc with Image lab software (Both from Bio-Rad, Hercules, CA, USA).

#### ASC oligomerization

ASC oligomers were stabilized by disuccinimidyl suberate (DSS) crosslinking as described in Khare et al. [81], with minor modifications. Cells were scraped in 200 μL lysis buffer consisting of 20 mM Hepes-KOH, pH 7.5, 150 mM KCL, 1% NP-40, 1 mM PMFS, 1 tablet Complete protease inhibitor cocktail (Roche, Basel, Switzerland) and 1 mM sodium ortovanadate. The protease inhibitor cocktail and PMFS were added just before cell lysis. Next, the cell suspensions were transferred to Eppendorf tubes and lysed by shearing 10 times through a 21-gauge needle. At this point, 50 μL cell lysate was removed to use as input control. The remaining lysate was centrifuged at 2700 x g for 10 min and the supernatant removed. The pellet was then resuspended in 500 μL PBS containing freshly prepared 2 mM DSS (Sigma, St. Louis, MO, USA) and incubated in a rotator for 30 min at room temperature. Following incubation, the cell lysates were centrifuged at 2700 x g to pellet the cross-linked proteins and the supernatant carefully removed. The pellet was then resuspended in 60 μL laemmli buffer.

#### TCA precipitation

Protein in the cell culture supernatant was precipitated using trichloroacetic acid (TCA) to increase the concentration in the samples for detection of cleaved caspase-1 and IL-1β. Equal volumes of sample and 20% TCA solution were incubated on ice for 30 min to precipitate the proteins. The samples were then centrifuged for 15 min at 12000 x g and the supernatant removed. Next, the samples were washed five times by adding 1 mL ice-cold acetone and centrifuging at 12000 x g for 10 min. After the final wash the supernatant was carefully removed and the pellets air-dried, before resuspending the pellet in 55 uL laemmeli sample buffer.

### Real-time quantitative polymerase chain reaction

RNA was isolated using the NucleoSpin RNA Plus kit (Macherey-Nage, Düren, Germany) and reverse transcribed into cDNA using a High-Capacity cDNA Reverse Transcription kit (Applied Biosystems by Thermo Fischer Scientific, Waltham, MA, USA) and a S-100 thermal cycler (BioRad, Hercules, CA, USA). Gene expression of CXCL8, IL-1β and GAPDH was determined by real-time quantitative PCR using a CFX96 Touch Real-Time PCR Detection System (BioRad, Hercules, CA, USA) with pre-designed TaqMan Gene Expression Assays and TaqMan Universal PCR Master Mix (Thermofischer, Waltham, MA, USA). The expression of each gene was normalized against the housekeeping gene GAPDH as described in Skuland et al. [59].

### Statistical analyses

Statistical analysis was performed using GraphPad Prism 8 (Version 8.0.1) or R (version 3.5.0). Statistically significant differences between exposed and control were determined using a two-way ANOVA with Dunnet’s or Sidak post-tests for multiple comparisons. One-way ANOVA and Tukey post-test were used for between particle comparisons of AUC values. Based on the evaluation of residual- and QQ plots, several data sets were log-transformed prior to analysis due to non-normality and heteroscedasticity. The associations between endpoints were assessed by linear regression analysis. *P* values below 0.05 were considered statistically significant.

AUC values were calculated for each experiment using the trapezoid rule. Cell viability data from the alamarBlue™ assay were normalized prior to calculating the AUC values by dividing each value by its respective control to adjust for differences in baseline levels. To adjust the AUC values to reflect exposure at an equal surface area, concentrations were first changed from μg/mL to m^2^/mL. Next, a curve was fitted to the data and new values were estimated from the portion of the curve corresponding to the concentrations common among all the particles. Based on the adjusted concentrations of quartzite, the particle sample with the lowest surface area, the concentration-range of 0–0.00152 m^2^/mL was used to compare responses at equal surface area concentrations. The equivalent mass-based concentrations when adjusted for differences in surface area were 0–400 μg/mL for quartzite, 0–211 μg/mL for anorthosite and rhomb porphyry, 0–292 μg/mL for dacite, 0–298 μg/mL for quartz diorite, and 0–217 μg/mL for α-quartz.

Due to inter-experimental variations in basal and particle-induced cytokine levels between the replicates in the NLRP3-inhibitor studies, values of each individual experiment were normalized against the mean response of all values in that experiment to better express the relative effect of the inhibitor MCC950.

## Supplementary Information

**Additional file 1: Figure S1.** Binding of cytokines to stone particles. 300 pg/mL CXCL8 (A), 200 pg/mL IL-1β (B), 300 pg/mL IL-1α (C) and 500 pg/mL TNFα (D) was incubated with 400 μg/mL of quartzite, anorthosite, rhomb porphyry, dacite, quartz diorite, hornfels and α-quartz in either RPMI (i) or DMEM (ii) medium. The levels of cytokines remaining in the medium after 24 h were measured with ELISA. Results are presented as mean ± SD of two independent experiments performed in triplicate.

**Additional file 2: Figure S2.** The effect of conditioned medium from particle-exposed cells in HBEC3-KT cells and THP-1 macrophages compared to a HBEC3-KT/THP-1 co-culture. HBEC3-KT cells, THP-1 macrophages and a co-culture of HBEC3-KT cells and THP-1 macrophages were exposed to 200 and 400 μg/mL α-quartz, or to the conditioned medium from particle-exposed HBEC3-KT and THP-1 for 24 h. The release of CXCL8 (A), IL-1β (B), IL-1α (C) and TNFα (D) in the cell culture supernatant was measured by ELISA. Results are presented as mean ± SD (*n* = 3–6).

**Additional file 3: Figure S3.** The association between cell viability and cytokine release in HBEC3-KT cells. Mean area under the curve (AUC) values were calculated for each particle sample from the data presented in Figs. 2 and 3.

**Additional file 4: Figure S4.** The association between cell viability and cytokine release in a HBEC3-KT/THP-1 co-culture Mean area under the curve (AUC) values were calculated for each particle sample from the data presented in Figs. 2 and 5.

**Additional file 5: Figure S5.** The association between cell viability and cytokine release in THP-1 macrophages. Mean area under the curve (AUC) values were calculated for each particle sample from the data presented in Figs. 2 and 4.

**Additional file 6: Figure S6.** The association between particle-induced hemolysis and cytokine release in HBEC3-KT cells. Mean area under the curve (AUC) values were calculated for each particle sample from the data presented in Figs. 3 and 6.

**Additional file 7: Figure S7.** Comparison of CXCL8 and IL-1β gene expression and cytokine release in HBEC3-KT cells. The cytokine release (white bars) and gene expression (grey bars) of CXCL8 (A) and IL-1β (B) in HBEC3-KT cells were determined after 24 and 12 h exposure to 200 μg/mL α-quartz, anorthosite, rhomb porphyry and quartz diorite, respectively. Gene expression was measured by real-time quantitative PCR, while cytokine release was measured using ELISA. Results are presented as mean ± SD (*n* = 4–7). Cytokine release data are also presented in Fig. 3.

**Additional file 8: Table S1.** Elemental composition of the stone particle samples.

## Data Availability

The datasets used in the presents study are available from the corresponding author upon reasonable request.

## Abbreviations

PM: Particulate matter
HBEC3-KT: Human bronchial epithelial cells
IL: Interleukin
CXCL8: Chemokine CXC-motif ligand 8
TNFα: Tumor necrosis factor alpha
NLRP3: Nucleotide-binding oligomerization domain (NOD)-like receptor containing pyrin domain 3
MSU: Monosodium urate
CPPD: Calcium pyrophosphate dehydrate
AP-1: Activator protein 1
NFkB: Nuclear factor kappa B
HBMG1: High mobility group box 1
ATP: Adenosine triphosphate
PMA: Phorbol myrisate acetate
DMSO: Dimethyl sulfoxide
AUC: Area under the curve
ASC: Apoptosis-associated speck-like protein containing a CARD
TCA: Trichloroacetic acid
