# Supplementary figures and images for: Respirable stone particles differ in their ability to induce cytotoxicity and pro-inflammatory responses in cell models of the human airways
Source: Part Fibre Toxicol. 2021 May 6;18:18. doi: 10.1186/s12989-021-00409-y (PMC8101231; doi:10.1186/s12989-021-00409-y)

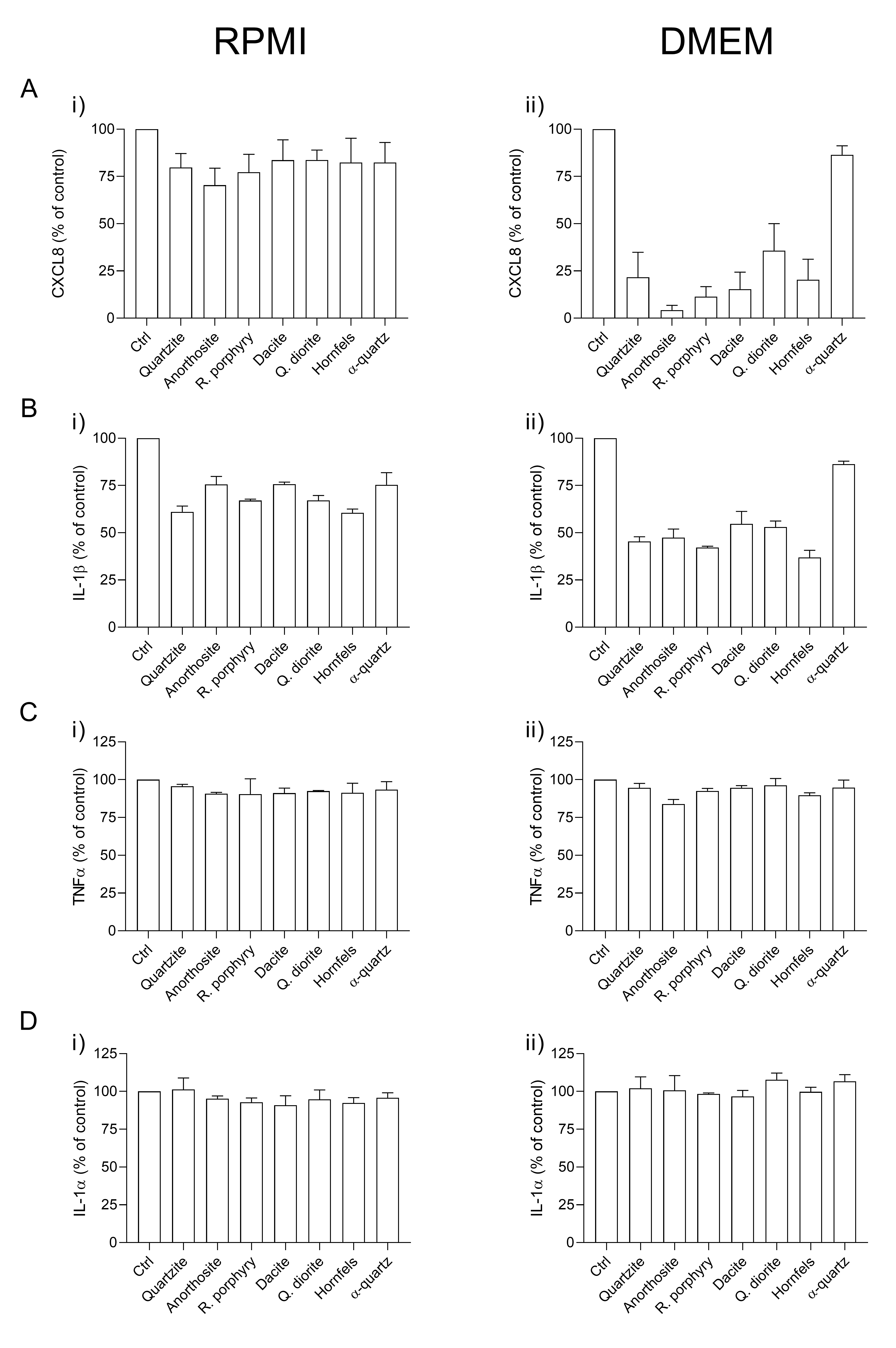

Supplement: Supplementary file 1 — Additional file 1: Figure S1. Binding of cytokines to stone particles. 300 pg/mL CXCL8 (A), 200 pg/mL IL-1β (B), 300 pg/mL IL-1α (C) and 500 pg/mL TNFα (D) was incubated with 400 μg/mL of quartzite, anorthosite, rhomb porphyry, dacite, quartz diorite, hornfels and α-quartz in either RPMI (i) or DMEM (ii) medium. The levels of cytokines remaining in the medium after 24 h were measured with ELISA. Results are presented as mean ± SD of two independent experiments performed in triplicate. [file 12989_2021_409_MOESM1_ESM.tif]

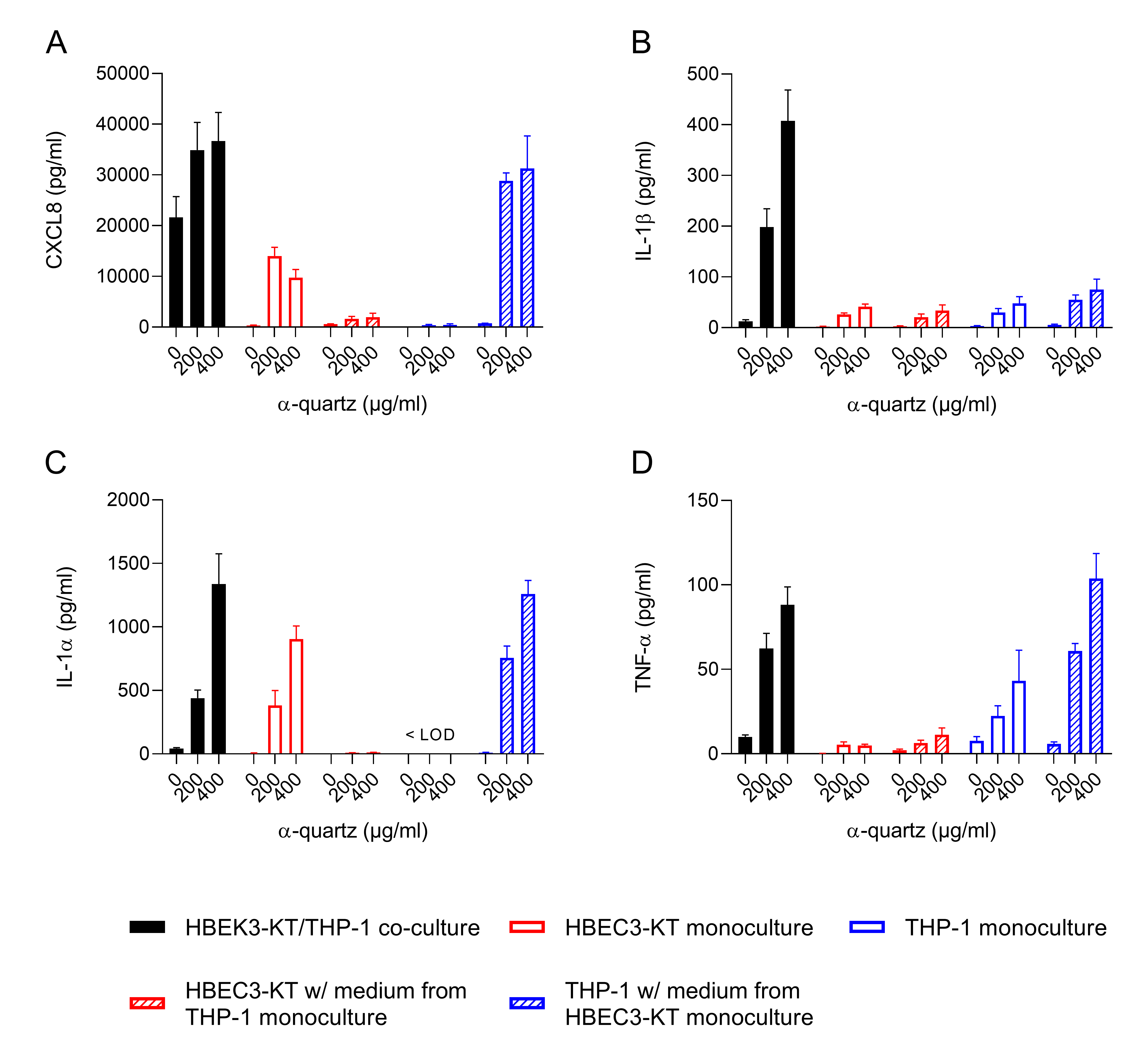

Supplement: Supplementary file 2 — Additional file 2: Figure S2. The effect of conditioned medium from particle-exposed cells in HBEC3-KT cells and THP-1 macrophages compared to a HBEC3-KT/THP-1 co-culture. HBEC3-KT cells, THP-1 macrophages and a co-culture of HBEC3-KT cells and THP-1 macrophages were exposed to 200 and 400 μg/mL α-quartz, or to the conditioned medium from particle-exposed HBEC3-KT and THP-1 for 24 h. The release of CXCL8 (A), IL-1β (B), IL-1α (C) and TNFα (D) in the cell culture supernatant was measured by ELISA. Results are presented as mean ± SD (n = 3–6). [file 12989_2021_409_MOESM2_ESM.tif]

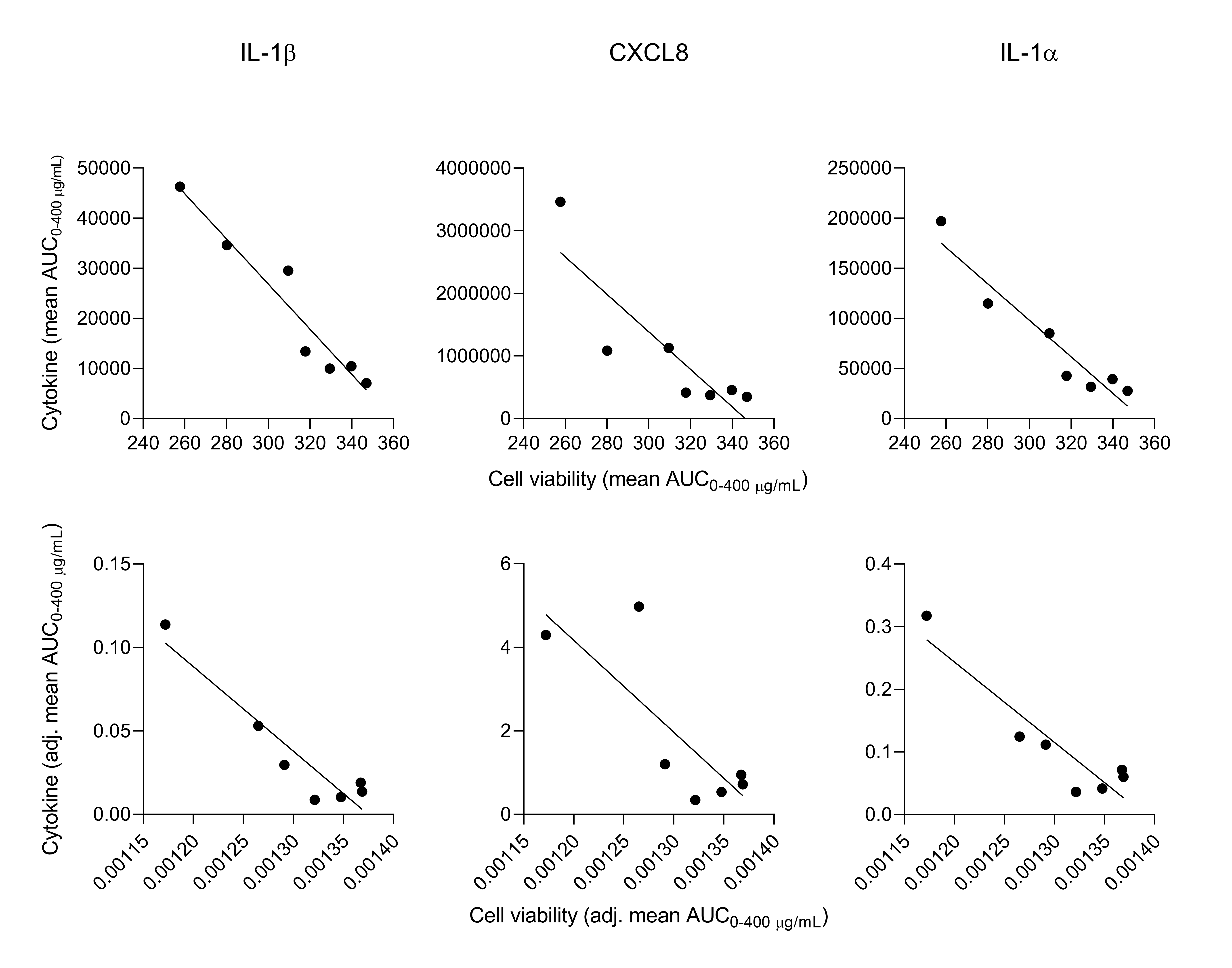

Supplement: Supplementary file 3 — Additional file 3: Figure S3. The association between cell viability and cytokine release in HBEC3-KT cells. Mean area under the curve (AUC) values were calculated for each particle sample from the data presented in Figs. 2 and 3. [file 12989_2021_409_MOESM3_ESM.tif]

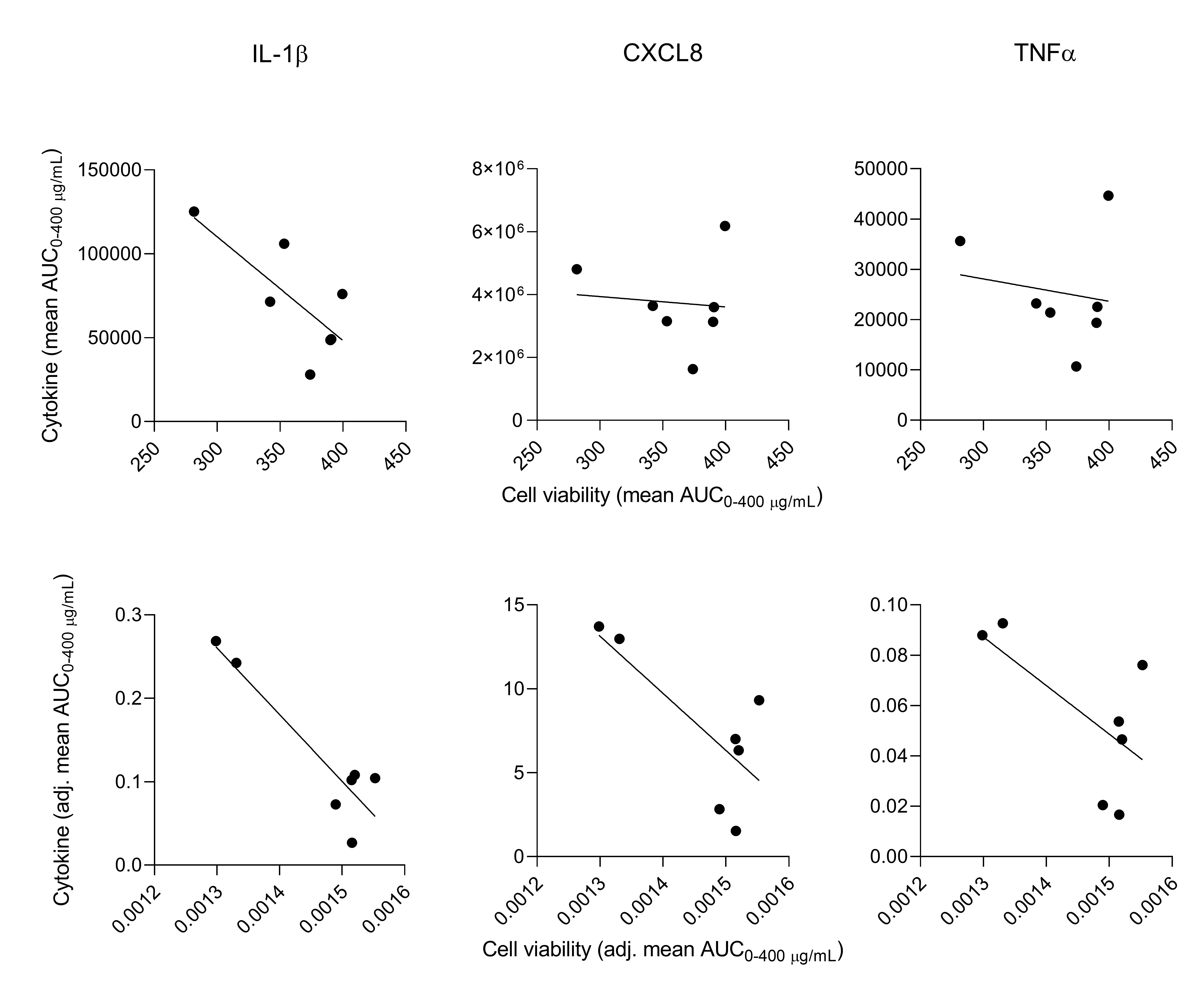

Supplement: Supplementary file 4 — Additional file 4: Figure S4. The association between cell viability and cytokine release in a HBEC3-KT/THP-1 co-culture Mean area under the curve (AUC) values were calculated for each particle sample from the data presented in Figs. 2 and 5. [file 12989_2021_409_MOESM4_ESM.tif]

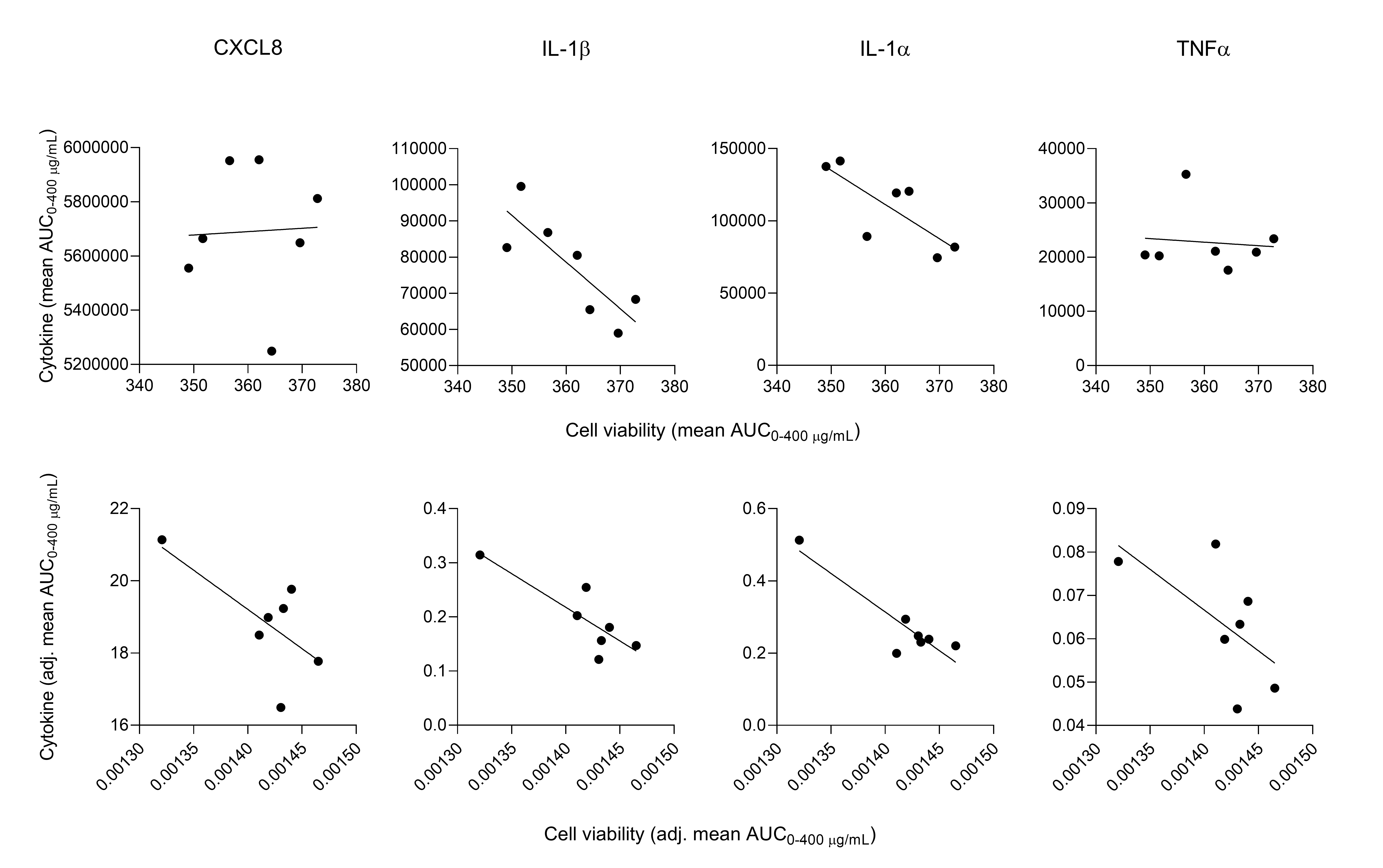

Supplement: Supplementary file 5 — Additional file 5: Figure S5. The association between cell viability and cytokine release in THP-1 macrophages. Mean area under the curve (AUC) values were calculated for each particle sample from the data presented in Figs. 2 and 4. [file 12989_2021_409_MOESM5_ESM.tif]

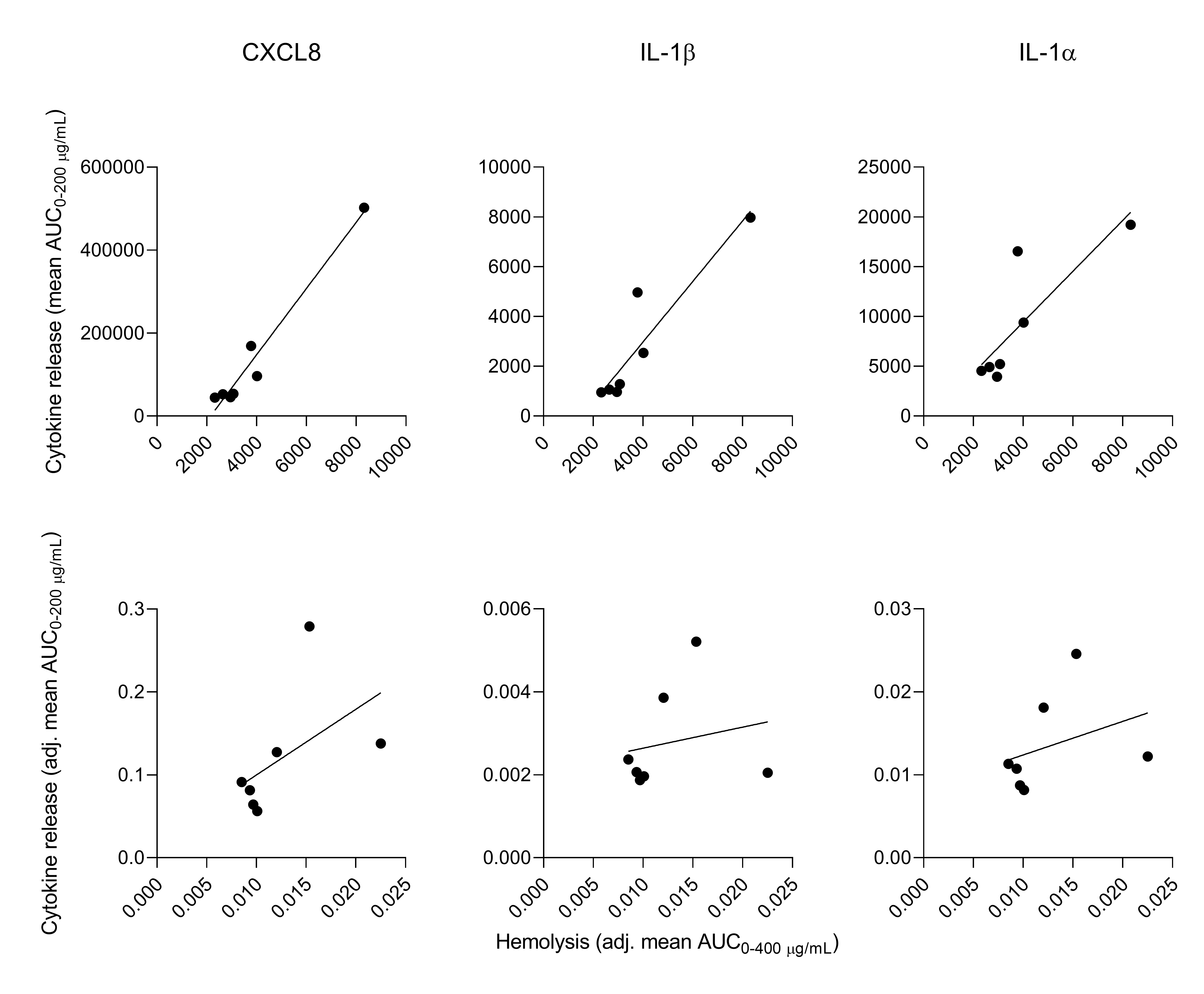

Supplement: Supplementary file 6 — Additional file 6: Figure S6. The association between particle-induced hemolysis and cytokine release in HBEC3-KT cells. Mean area under the curve (AUC) values were calculated for each particle sample from the data presented in Figs. 3 and 6. [file 12989_2021_409_MOESM6_ESM.tif]

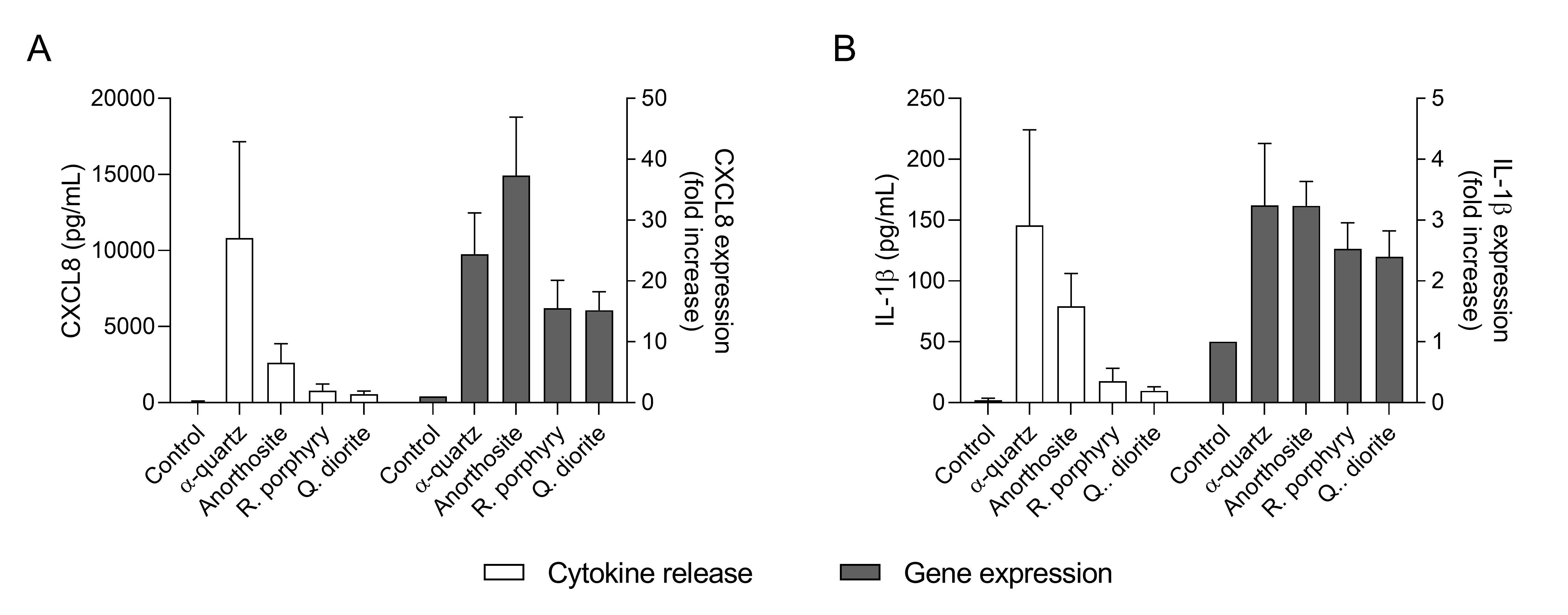

Supplement: Supplementary file 7 — Additional file 7: Figure S7. Comparison of CXCL8 and IL-1β gene expression and cytokine release in HBEC3-KT cells. The cytokine release (white bars) and gene expression (grey bars) of CXCL8 (A) and IL-1β (B) in HBEC3-KT cells were determined after 24 and 12 h exposure to 200 μg/mL α-quartz, anorthosite, rhomb porphyry and quartz diorite, respectively. Gene expression was measured by real-time quantitative PCR, while cytokine release was measured using ELISA. Results are presented as mean ± SD (n = 4–7). Cytokine release data are also presented in Fig. 3. [file 12989_2021_409_MOESM7_ESM.tif]
