# Supplementary material for: Respirable stone particles differ in their ability to induce cytotoxicity and pro-inflammatory responses in cell models of the human airways
Source: Part Fibre Toxicol. 2021 May 6;18:18. doi: 10.1186/s12989-021-00409-y (PMC8101231; doi:10.1186/s12989-021-00409-y)
Supplement: Supplementary file 8 — Additional file 8: Table S1. Elemental composition of the stone particle samples. [file 12989_2021_409_MOESM8_ESM.docx]

**Table S1.** **Elemental composition of the stone particle samples.**

|  | Quartzite | Anorthosite | R. porphyry | Dacite | Q. diorite | Hornfels |
| --- | --- | --- | --- | --- | --- | --- |
| SiO_2_ | 86.800 | 54.400 | 58.700 | 63.600 | 59.900 | 60.600 |
| Al_2_O_3_ | 6.950 | 26.600 | 17.600 | 18.700 | 15.900 | 15.100 |
| Fe_2_O_3_ | 0.981 | 1.320 | 3.570 | 3.330 | 6.680 | 4.790 |
| TiO_2_ | 0.247 | 0.198 | 1.030 | 0.304 | 0.744 | 0.750 |
| MgO | 0.290 | 0.863 | 1.900 | 1.030 | 3.750 | 2.550 |
| CaO | 0.425 | 4.880 | 2.590 | 3.890 | 4.030 | 6.170 |
| Na_2_O | 0.190 | 6.210 | 4.770 | 5.140 | 3.170 | 2.520 |
| K_2_O | 1.550 | 1.800 | 5.460 | 1.260 | 2.210 | 4.630 |
| MnO | 0.014 | 0.020 | 0.108 | 0.071 | 0.121 | 0.088 |
| P_2_O_5_ | 0.061 | 0.056 | 0.233 | 0.070 | 0.119 | 0.057 |
| BaO | 0.021 | 0.038 | 0.104 | 0.048 | 0.056 | 0.061 |
| Co_2_O_3_ | <0.004 | <0.004 | <0.004 | <0.004 | <0.004 | <0.004 |
| Cr_2_O_3_ | <0.007 | <0.007 | <0.007 | <0.007 | <0.007 | 0.029 |
| CuO | <0.01 | <0.01 | <0.01 | <0.01 | 0.010 | <0.01 |
| NiO | <0.01 | <0.01 | <0.01 | <0.01 | <0.01 | 0.009 |
| PbO | <0.01 | <0.01 | <0.01 | <0.01 | <0.01 | <0.01 |
| SrO | 0.014 | 0.086 | 0.069 | 0.071 | 0.043 | 0.070 |
| V_2_O_3_ | <0.007 | <0.007 | <0.007 | <0.007 | 0.014 | 0.014 |
| ZnO | <0.01 | <0.01 | 0.009 | 0.009 | 0.016 | 0.012 |
| ZrO_2_ | 0.019 | <0.007 | 0.357 | <0.007 | 0.021 | 0.025 |
